# Comparative Physicochemical Characterization and Biological Effects of Magnetite- and Maghemite-Based Magnetic Colloidal Suspensions in A704 Renal Cancer Cells

**DOI:** 10.3390/ph19091381

**Published:** 2026-09-01

**Authors:** Mihai Cristian Neagu, Diana Haj Ali, Emil Radu Iacob, Andreea Smeu, Roxana Stoicescu, Călin Marius Popoiu, Georgiana Boştinaru, Ştefan Marcu, Robert Ianoş, Vlad Socoliuc, Lucian Barbu Tudoran, Elena-Alina Moacă

**Affiliations:** 1 Doctoral School of Medicine, “Victor Babeş” University of Medicine and Pharmacy Timisoara, 2 Eftimie Murgu Square, 300041 Timisoara, Romania; mihai.neagu@umft.ro; 2 University Clinic of Pediatric Surgery and Orthopedics, Faculty of Medicine, “Victor Babes” University of Medicine and Pharmacy Timisoara, 2 Eftimie Murgu Square, 300041 Timisoara, Romania; mcpopoiu@umft.ro (C.M.P.); georgiana.trailescu@umft.ro (G.B.); marcu.stefan@umft.ro (Ş.M.); 3 Center for Multidisciplinary Research and Management in Pediatric Surgical Conditions (CCMMA-CHIR-PED), 2 Iosif Nemoianu Street, 300011 Timisoara, Romania; 4 University Clinic of Toxicology, Drug Industry, Management, Marketing and Dermatopharmacy, Faculty of Pharmacy, “Victor Babeș” University of Medicine and Pharmacy Timisoara, 2 Eftimie Murgu Square, 300041 Timisoara, Romania; diana.haj-ali@umft.ro (D.H.A.); alina.moaca@umft.ro (E.-A.M.); 5 Research Centre for Pharmaco-Toxicological Evaluations, “Victor Babeș” University of Medicine and Pharmacy Timisoara, 2 Eftimie Murgu Square, 300041 Timisoara, Romania; 6 Department of Anatomy and Embryology, Faculty of Medicine, “Victor Babeş” University of Medicine and Pharmacy Timisoara, 2 Eftimie Murgu Square, 300041 Timisoara, Romania; roxana.iacob@umft.ro; 7 Research Center for Medical Communication, “Victor Babeș” University of Medicine and Pharmacy Timisoara, 2 Eftimie Murgu Square, 300041 Timisoara, Romania; 8 Faculty of Chemical Engineering, Biotechnologies and Environmental Protection, Politehnica University Timisoara, 6 Vasile Pârvan Blvd., 300223 Timisoara, Romania; robert_ianos@yahoo.com; 9 Laboratory of Magnetic Fluids, Center for Fundamental and Advanced Technical Research, Romanian Academy—Timisoara Branch, 24 M. Viteazu Ave., 300223 Timisoara, Romania; vsocoliuc@gmail.com; 10 Research Center for Complex Fluids Systems Engineering, Politehnica University of Timisoara, 1 M. Viteazu Ave., 300222 Timisoara, Romania; 11 Electron Microscopy Laboratory “Prof. C. Craciun”, Faculty of Biology & Geology, “Babes-Bolyai” University, 5-7 Clinicilor Street, 400006 Cluj-Napoca, Romania; lucianbarbu@yahoo.com; 12 Electron Microscopy Integrated Laboratory, National Institute for R&D of Isotopic and Molecular Technologies, 67-103 Donat Street, 400293 Cluj-Napoca, Romania

**Keywords:** magnetic iron oxide nanoparticles, magnetic colloidal suspensions, magnetite, maghemite, oleic acid coating, renal cell carcinoma, A704 cells, mitochondrial membrane potential, cytotoxicity, clonogenic capacity

## Abstract

**Background/Objectives**: Magnetic iron oxide nanoparticles are investigated in cancer research; however, the direct biological effects of unloaded magnetic colloidal suspensions in renal cancer remain insufficiently characterized. This study compared two double-oleic-acid-coated formulations prepared from precursors: magnetite-based MCS 1 and maghemite-based MCS 2. **Methods**: The suspensions were characterized by vibrating-sample magnetometry, dynamic light scattering, bright-field scanning transmission electron microscopy, and energy-dispersive X-ray spectroscopy. Their effects on A704 renal adenocarcinoma cells were evaluated after 24 h of exposure to 1–5 µg/mL using MTT, neutral red uptake, JC-1, Hoechst 33342/MitoTracker Red CMXRos, acridine orange/propidium iodide staining, and a 7-day clonogenic assay. **Results**: MCS 2 showed higher volumetric saturation magnetization (1.541 vs. 1.059 Gs), a smaller Z-average hydrodynamic diameter (79.65 vs. 103.9 nm), and a more uniform volume-weighted distribution. Both formulations significantly reduced metabolic activity and neutral red uptake and induced mitochondrial depolarization, cell-death-associated morphological changes, and impaired clonogenic capacity. MCS 1 generally produced a moderate response with an apparent plateau, whereas MCS 2 showed a pronounced concentration-related effect at 4–5 µg/mL. At 5 µg/mL, MCS 2 reduced metabolic activity to approximately 49%, neutral red uptake to 50.04%, the JC-1 aggregate-to-monomer ratio to 46.02%, and colony formation to 31.44% of the control. **Conclusions**: The suspensions exhibited distinct physicochemical and biological profiles. MCS 2 produced greater effects at the highest concentrations, potentially related to its smaller hydrodynamic size and more uniform particle-size distribution. Further studies should establish tumor selectivity, cellular uptake, and the underlying molecular mechanisms.

## 1. Introduction

Renal cell carcinoma (RCC) represents the predominant form of malignant kidney tumor in adults and remains a clinically challenging and biologically heterogeneous malignancy, particularly in advanced disease [1,2]. Despite substantial progress in systemic treatment, therapeutic resistance, disease progression, and treatment-related adverse effects continue to motivate the investigation of complementary experimental approaches [2,3,4,5]. In the present study, A704 human renal adenocarcinoma cells were employed as an experimental cancer-cell model for the comparative biological assessment of two distinct magnetic colloidal formulations.

Nanotechnology offers opportunities to develop multifunctional systems capable of improving tumour localization, imaging, drug delivery, and externally controlled therapeutic interventions. Among the investigated nanomaterials, magnetic iron oxide nanoparticles (MIONPs), particularly those containing magnetite (Fe_3_O_4_) or maghemite (γ-Fe_2_O_3_), are of considerable interest because of their magnetic responsiveness, comparatively large surface area, and amenability to surface modification. These properties have supported their investigation as magnetic resonance imaging contrast agents, drug-delivery vehicles, magnetic targeting systems, hyperthermia mediators, and mechanically actuated therapeutic particles [6,7,8,9]. The biological performance of MIONPs is not determined exclusively by the chemical identity of the iron oxide core. Particle size, crystallinity, morphology, surface area, hydrodynamic diameter, agglomeration state, surface coating, and colloidal behaviour in biological media can markedly influence cellular association, uptake, intracellular trafficking, and toxicity [7,10]. Smaller particles generally provide a larger accessible surface area per unit mass, although their biological activity also depends on coating chemistry and the formation of particle aggregates or protein coronas. Therefore, physicochemical characterization of the final colloidal formulation, rather than characterization of the precursor powder alone, is essential when interpreting its biological effects. Surface modification is particularly important because uncoated iron oxide nanoparticles tend to interact and aggregate in aqueous or physiological environments. Oleic acid has a strong affinity for iron oxide surfaces and is frequently employed to control nanoparticle growth and reduce direct core–core interactions. The formation of an oleic acid bilayer can facilitate the transfer and stabilization of iron oxide nanoparticles in aqueous media [11,12]. However, the resulting hydrodynamic entities may be substantially larger than the inorganic cores because the DLS-derived diameter includes the organic coating, associated solvent layer, and any particulate assemblies present in suspension.

The iron oxide nanoparticles should not necessarily be considered biologically inert carriers. Depending on their physicochemical characteristics and surface chemistry, internalized particles may undergo endosomal and lysosomal trafficking, intracellular transformation, and interactions with redox-active cellular pathways. Experimental studies have associated selected iron oxide formulations with reactive oxygen species generation, mitochondrial membrane depolarization, cell-cycle alterations, autophagic responses, and apoptosis [7,13,14]. In human hepatoma cells, both uncoated and oleic-acid-coated Fe_3_O_4_ nanoparticles produced concentration-dependent cytotoxicity accompanied by mitochondrial alterations and apoptosis-associated molecular changes [13]. Mitochondria-targeted iron oxide nanoparticles have also been shown to undergo intracellular chemical transformation and to induce pronounced mitochondrial depolarization in breast cancer cells [14]. Conversely, other formulations have displayed limited cytotoxicity, demonstrating that the biological response is strongly formulation- and cell-type-dependent [7].

Magnetic nanoparticle-based strategies have also been investigated specifically in renal cancer models. Shinkai et al. developed G250 antibody-targeted magnetoliposomes that accumulated in renal carcinoma tissue and inhibited tumour growth following exposure to an alternating magnetic field [8]. Leulmi et al. demonstrated apoptosis in targeted human renal cancer cells through low-frequency vibration of anisotropic magnetic particles attached to the plasma membrane [9]. More recently, magnetic-core poly(lactic-co-glycolic acid) (PLGA) carriers loaded with silibinin were evaluated in A-498 kidney cancer cells and produced greater cytotoxic effects than free silibinin [15]. These studies demonstrate the therapeutic potential of magnetic systems in renal malignancies; however, the representative approaches have primarily relied on drug loading, tumour-specific functionalization, magnetic hyperthermia, or external magnetic actuation.

The direct biological effects of unloaded aqueous magnetic colloidal suspensions containing double-oleic-acid-coated magnetite or maghemite nanoparticles remain less well characterized in renal cancer cells. In particular, comparative information linking the properties of structurally distinct precursor nanopowders and the characteristics of the resulting colloidal suspensions to their biological activity is limited. Such comparisons may help determine whether differences in crystalline phase, primary particle dimensions, specific surface area, magnetic behaviour, and hydrodynamic organization are associated with distinct cellular responses. The novelty of the present study lies in the integrated, side-by-side evaluation of two physicochemically distinct magnetic colloidal formulations and in relating their precursor and formulation-level characteristics to their biological responses. Unlike magnetic nanoparticle-based approaches relying on drug loading or externally applied magnetic stimulation, the present work examines the intrinsic biological effects of unloaded, double-oleic-acid-coated magnetite- and maghemite-based colloidal suspensions using complementary short- and long-term cellular endpoints.

Therefore, the present study aimed to prepare and comparatively characterize two aqueous magnetic colloidal suspensions based on double-oleic-acid-coated MIONPs obtained from structurally distinct precursor powders: a magnetite-based formulation, designated MCS 1, and a maghemite-based formulation, designated MCS 2. The magnetic response, hydrodynamic size distribution, morphology, and local elemental composition of the final suspensions were examined. Their in vitro biological effects were subsequently evaluated in A704 human renal adenocarcinoma cells using complementary assessments of cellular metabolic activity, neutral red uptake, bright-field morphology, mitochondrial membrane potential, nuclear and mitochondrial staining patterns, cell-death-associated morphology, and long-term clonogenic capacity. The study was designed to determine whether the distinct physicochemical profiles of the two formulations were associated with different cellular responses in the absence of drug loading or externally applied magnetic stimulation.

## 2. Results

### 2.1. Physicochemical Characterization of MCS 1 and MCS 2

**Figure 1:**
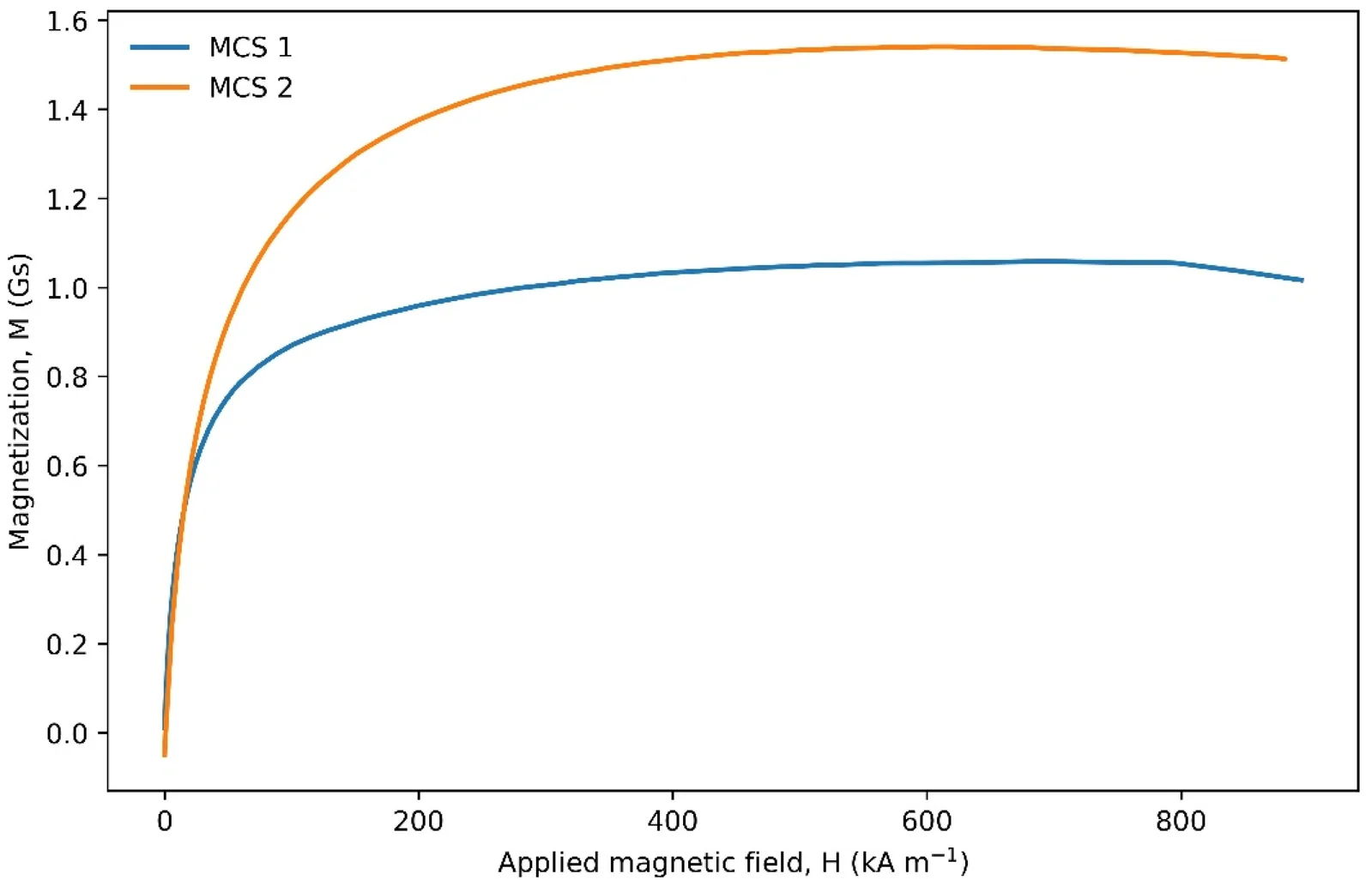
Virgin magnetization curves of MCS 1 and MCS 2 as a function of the applied magnetic field. Magnetization is expressed in gauss (Gs), whereas the applied magnetic field is expressed in kA/m.

The virgin magnetization curves of MCS 1 and MCS 2 exhibited a rapid increase in magnetization at low applied magnetic fields, followed by a progressive approach to saturation at higher field strengths (Figure 1). The saturation magnetization values extracted from the VSM measurements were 1.059 Gs for MCS 1 and 1.541 Gs for MCS 2.

Thus, the volumetric saturation magnetization of MCS 2 was approximately 45.5% higher than that of MCS 1. The magnetic field values required to reach 95% of the corresponding saturation magnetization were 301.9 kA/m for MCS 1 and 295.5 kA/m for MCS 2, indicating comparable approaches to magnetic saturation. Based on the saturation magnetization values and an effective magnetization of the dispersed magnetic solid phase of 4.29 × 10^3^ Gs, the calculated magnetic-particle volume fractions were 2.47 × 10^−4^ for MCS 1 and 3.59 × 10^−4^ for MCS 2. Using the theoretical densities of magnetite and maghemite, the corresponding mass concentrations of the magnetic phases were calculated as 1.284 mg Fe_3_O_4_/mL for MCS 1 and 1.757 mg γ-Fe_2_O_3_/mL for MCS 2. These calculated concentrations were subsequently used to prepare the working concentrations employed in the biological experiments.

**Figure 2:**
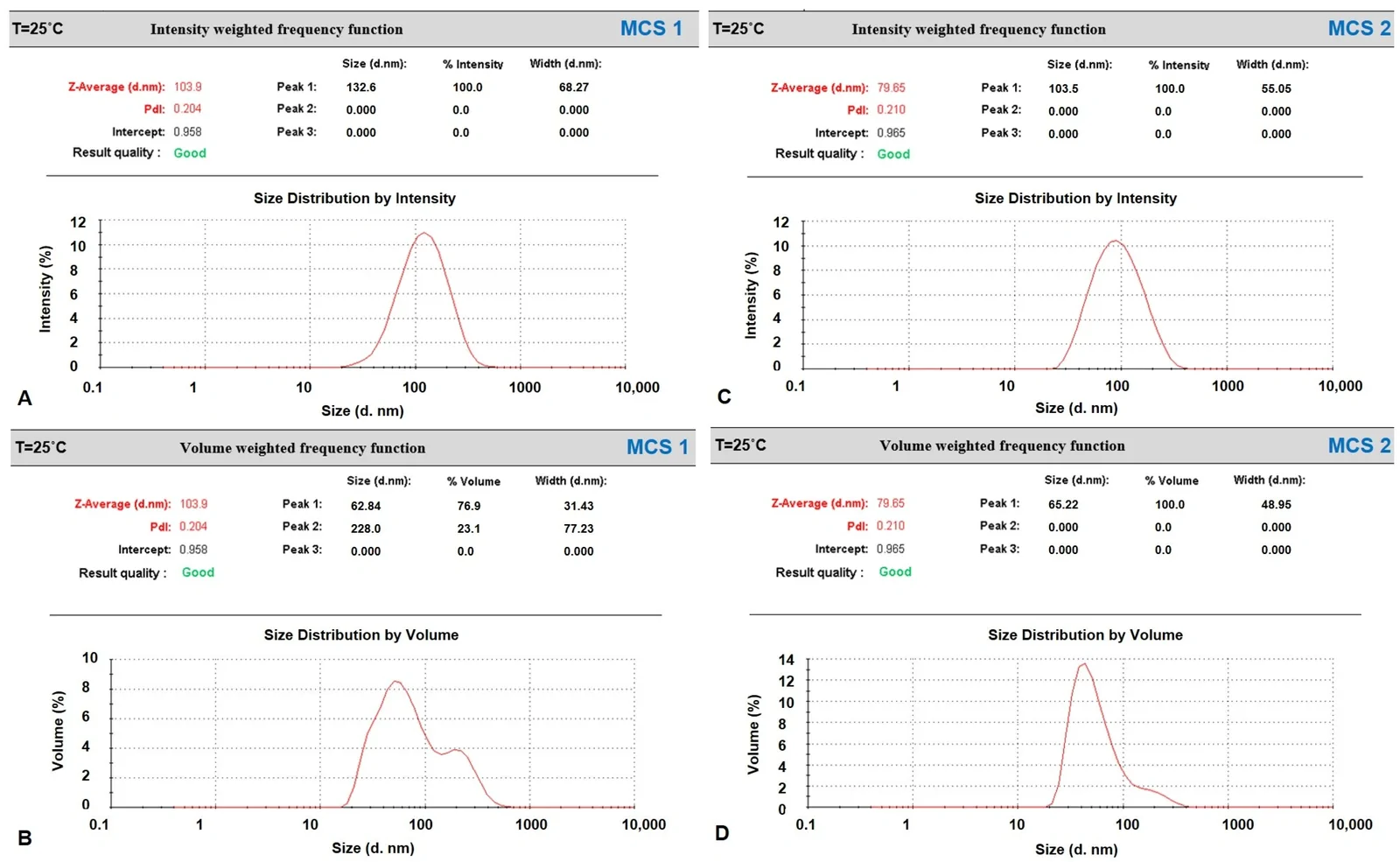
Dynamic light scattering analysis of MCS 1 and MCS 2 at 25 °C. Intensity-weighted particle size distributions of MCS 1 (**A**) and MCS 2 (**C**), and the corresponding volume-weighted distributions of MCS 1 (**B**) and MCS 2 (**D**). The Z-average hydrodynamic diameter, polydispersity index (PDI), intercept, peak position, relative contribution, and distribution width are displayed in the original instrument-generated reports.

Dynamic light scattering measurements performed at 25 °C revealed differences in the hydrodynamic size distributions of the two magnetic colloidal suspensions (Figure 2). MCS 1 exhibited a Z-average hydrodynamic diameter of 103.9 nm and a polydispersity index (PDI) of 0.204 (Figure 2A,B), whereas MCS 2 showed a smaller Z-average diameter of 79.65 nm and a comparable PDI of 0.210 (Figure 2C,D). The corresponding intercept values were 0.958 and 0.965, respectively, and both measurements were classified by the instrument software as having good result quality.

The intensity-weighted distributions showed a single dominant population for both formulations, centered at 132.6 nm for MCS 1 (Figure 2A) and 103.5 nm for MCS 2 (Figure 2C). The volume-weighted distribution of MCS 1 (Figure 2B) revealed a predominant population centered at 62.84 nm, accounting for 76.9% of the total volume, together with a secondary population centered at 228.0 nm, accounting for 23.1%. In contrast, MCS 2 (Figure 2D) displayed a single volume-weighted population centered at 65.22 nm. Overall, these findings indicate that MCS 2 had a smaller mean hydrodynamic diameter and a more uniform volume-weighted size distribution, whereas MCS 1 contained a minor fraction of larger hydrodynamic entities, possibly corresponding to particle aggregates.

**Figure 3:**
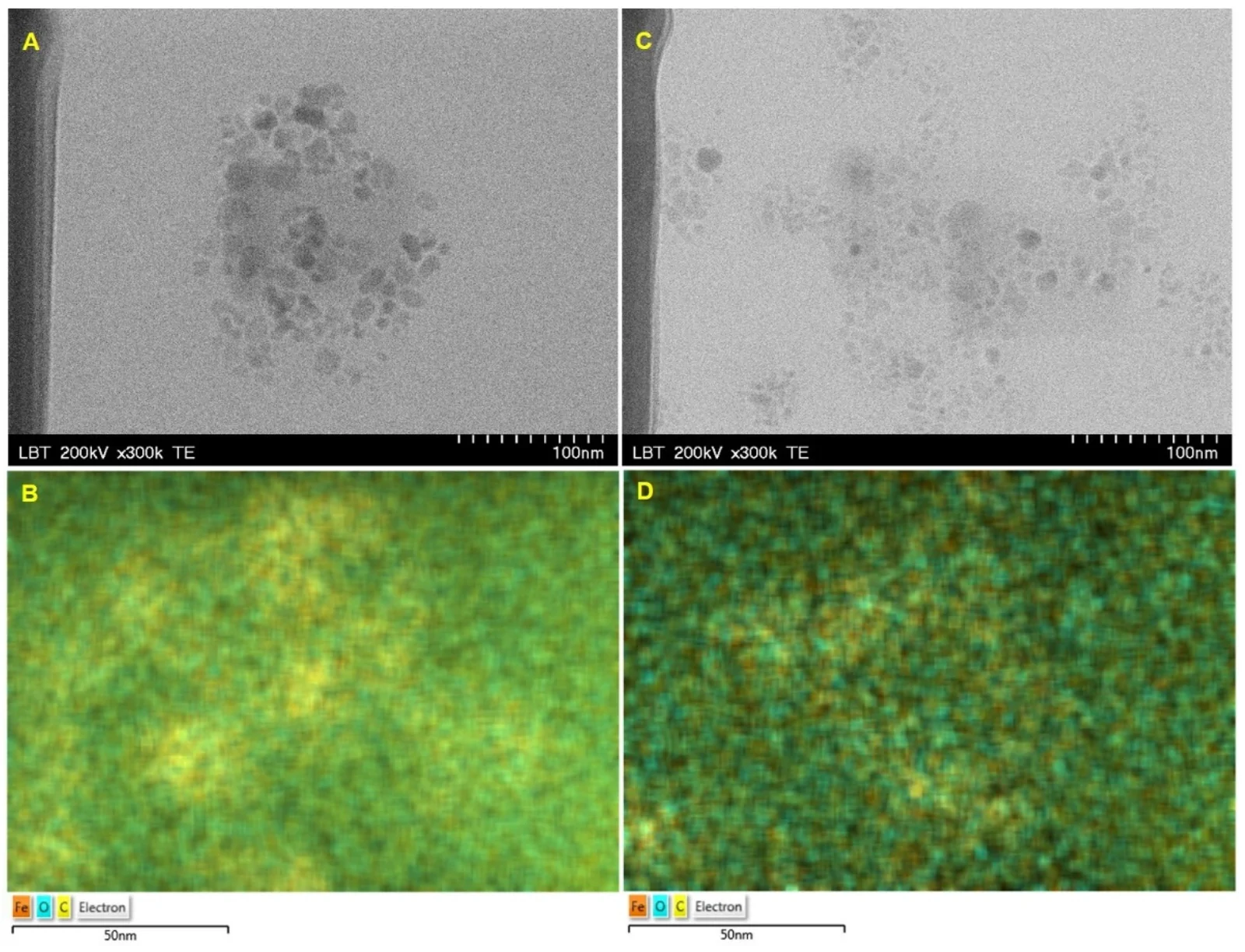
Representative BF-STEM images and EDX elemental maps of the magnetic nanoparticles dispersed in MCS 1 and MCS 2. (**A**) BF-STEM image of MCS 1; (**B**) combined EDX elemental map of MCS 1; (**C**) BF-STEM image of MCS 2; and (**D**) combined EDX elemental map of MCS 2. The BF-STEM images were acquired at an accelerating voltage of 200 kV and a magnification of ×300,000. Scale bars: 100 nm for BF-STEM images and 50 nm for the combined EDX elemental map. In the elemental maps, Fe, O, and C are displayed in orange, cyan, and yellow, respectively.

BF-STEM examination revealed morphological differences between the nanoparticles dispersed in the two magnetic colloidal suspensions (Figure 3). Sample MCS 1 contained predominantly quasi-spherical to slightly polyhedral particles arranged in relatively compact and irregular aggregates (Figure 3A). In contrast, sample MCS 2 consisted of apparently finer and more numerous quasi-spherical particles distributed within comparatively diffuse particulate assemblies (Figure 3C). These observations are consistent with the smaller crystallite and BET-derived particle dimensions previously reported for the MIONPs_2 precursor and with the lower hydrodynamic diameter of sample MCS 2 determined by DLS.

EDX analysis confirmed the presence and spatial association of Fe and O within the nanoparticle-containing regions of both samples, supporting the iron oxide nature of the dispersed nanoparticles. The local semi-quantitative elemental composition is summarized in Table 1. Because the measurements were obtained from selected microscopic regions and were influenced by the amount of deposited material and the microscopy support, differences between the elemental weight percentages of MCS 1 and MCS 2 were not interpreted as representative of their bulk composition.

### 2.2. In Vitro Biological Effects of MCS 1 and MCS 2 on A704 Renal Cancer Cells

#### 2.2.1. Effects on A704 Cell Metabolic Activity

**Figure 4:**
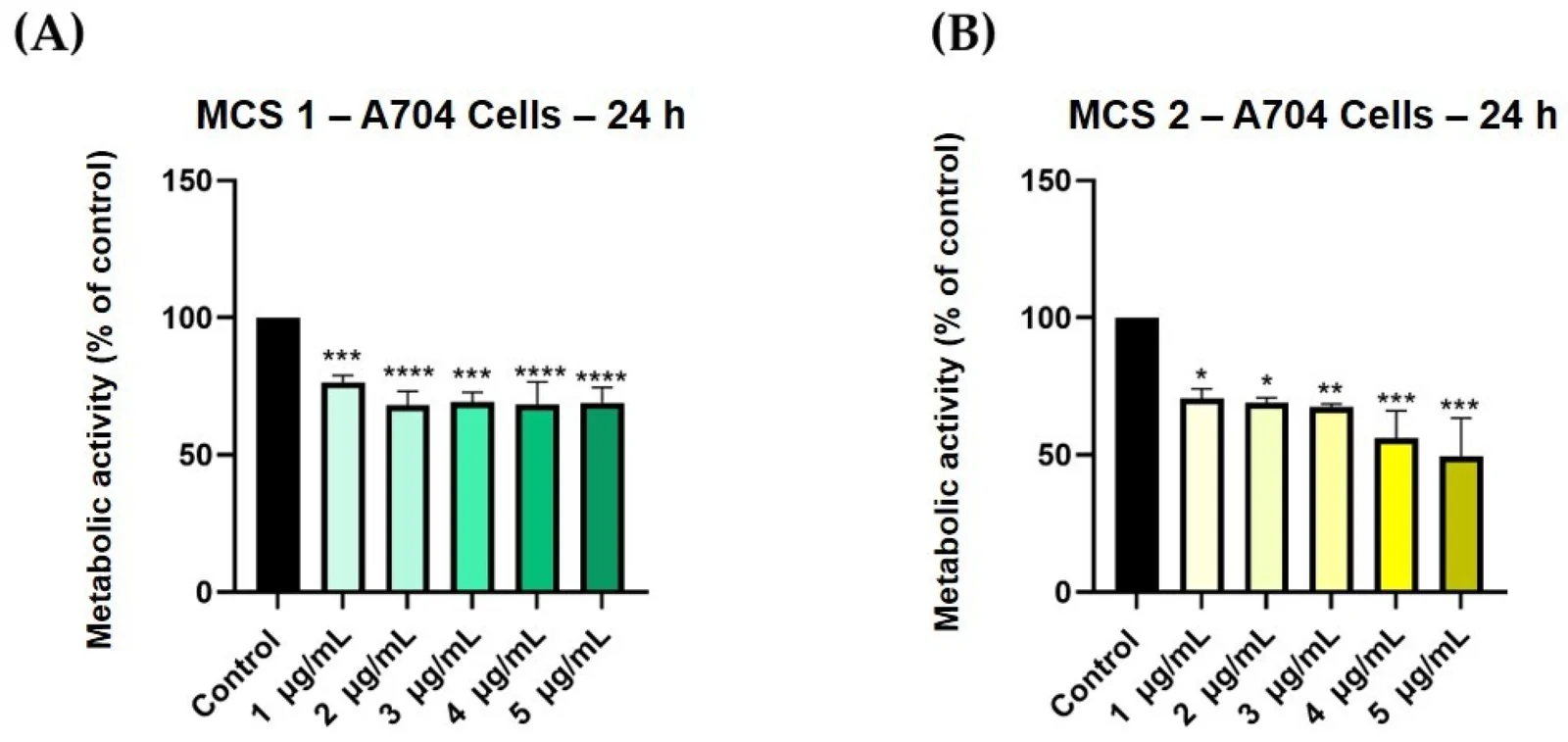
Effects of MCS 1 (**A**) and MCS 2 (**B**) samples on the metabolic activity of A704 human renal adenocarcinoma cells after 24 h of exposure to concentrations of 1, 2, 3, 4, and 5 µg/mL. Results were normalized to the untreated control, which was considered 100%, and are presented as mean ± standard deviation of three independent experiments. Statistical significance versus the untreated control was determined separately for each formulation using one-way ANOVA followed by Dunnett’s multiple-comparisons test. Statistical significance is indicated as follows: * *p* < 0.05; ** *p* < 0.01; *** *p* < 0.001; and **** *p* < 0.0001.

The effects of MCS 1 and MCS 2 on the metabolic activity of A704 cells were evaluated after 24 h of exposure using the MTT assay (Figure 4). Both magnetic colloidal suspensions significantly reduced cellular metabolic activity relative to the untreated control across the tested concentration range.

MCS 1 decreased metabolic activity to approximately 76% of the control at 1 µg/mL. A further reduction to approximately 68–69% was observed at concentrations of 2–5 µg/mL. The response remained relatively constant throughout this concentration range, suggesting that the effect of MCS 1 reached an apparent plateau rather than following a progressive concentration-dependent pattern. A greater degree of variability was observed at 4 µg/mL, as indicated by the larger standard deviation.

In contrast, MCS 2 produced a more pronounced concentration-related reduction in metabolic activity. Values remained at approximately 67–71% of the control following exposure to 1–3 µg/mL, whereas a stronger effect was observed at the two highest concentrations. Metabolic activity decreased to approximately 56% at 4 µg/mL and 49% at 5 µg/mL, corresponding to a reduction of approximately 51% relative to the untreated control at the highest tested concentration.

Overall, MCS 2 produced numerically stronger effects than MCS 1 at concentrations of 4 and 5 µg/mL. However, because the statistical analysis was performed by comparing each treatment group with its corresponding untreated control, differences between the two formulations were interpreted descriptively and not as direct statistically significant comparisons.

#### 2.2.2. Treatment-Associated Changes in Cellular Morphology

**Figure 5:**
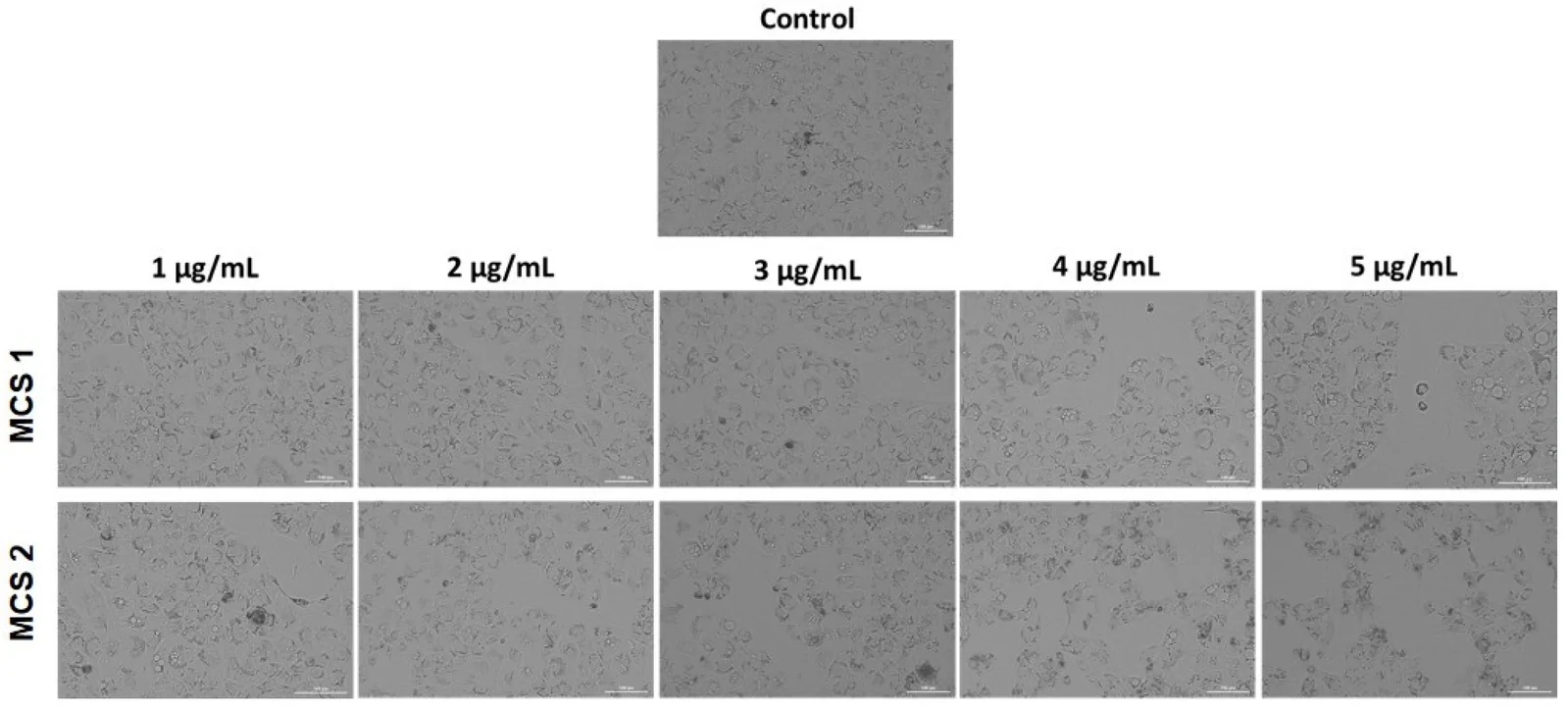
Representative bright-field images of A704 human renal adenocarcinoma cells following 24 h of exposure to MCS 1 and MCS 2 at concentrations of 1, 2, 3, 4, and 5 µg/mL. Untreated cells served as the control. Images were acquired at 20× magnification. Scale bar: 100 µm.

Bright-field microscopy was used to examine treatment-associated changes in A704 cell morphology after 24 h of exposure to MCS 1 and MCS 2 samples (Figure 5). Untreated cells displayed the typical adherent morphology of A704 cultures, with a relatively high cell density and preserved cellular shape.

Following exposure to MCS 1, the overall cellular morphology remained largely preserved across the tested concentration range. A modest reduction in cell density and confluence was observed, particularly at the higher concentrations, but no marked morphological alterations were evident. In contrast, MCS 2 produced a more pronounced concentration-related reduction in cell density and confluence. At concentrations of 4 and 5 µg/mL, the cultures contained a greater proportion of rounded and shrunken cells, together with reduced cellular spreading and the presence of cellular debris. These qualitative morphological changes were more evident than those observed following MCS 1 treatment and were consistent with the stronger reduction in metabolic activity detected for MCS 2 at the highest tested concentrations.

#### 2.2.3. Effects of MCS 1 and MCS 2 on Neutral Red Uptake

**Figure 6:**
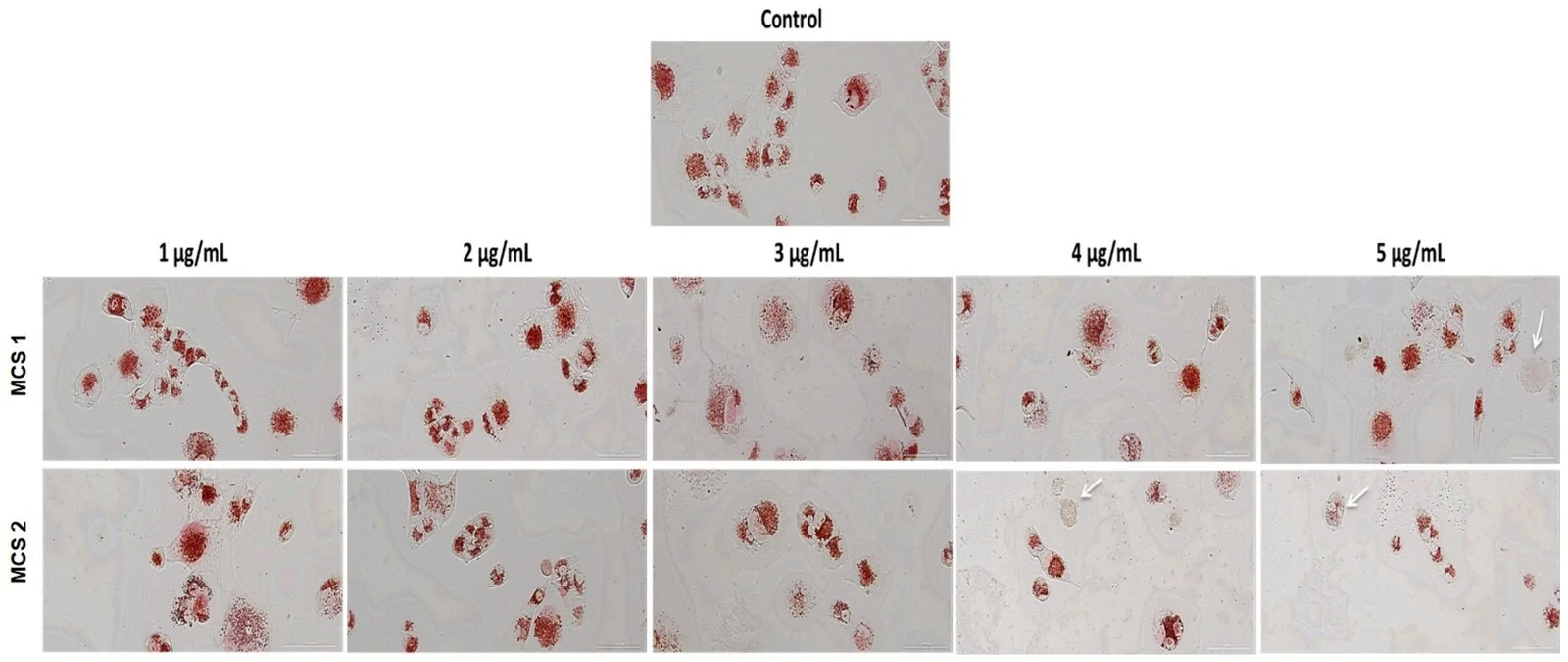
Representative bright-field images of neutral red staining in A704 human renal adenocarcinoma cells following 24 h of exposure to MCS 1 and MCS 2 at concentrations of 1, 2, 3, 4, and 5 µg/mL. Untreated cells served as the control. White arrows indicate representative areas with visibly reduced intracellular neutral red staining. Images were acquired at 20× magnification. Scale bar: 100 µm.

**Figure 7:**
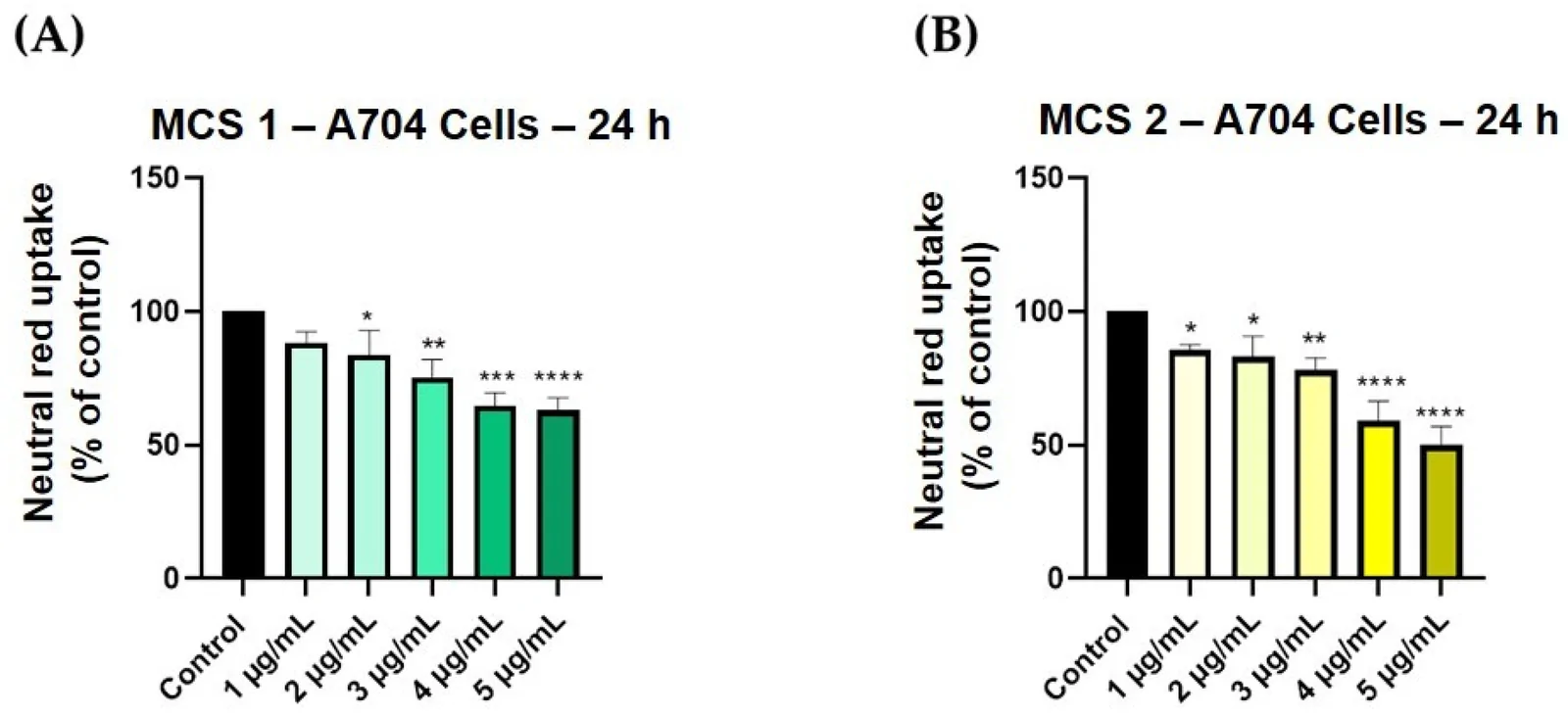
Effects of MCS 1 (**A**) and MCS 2 (**B**) on neutral red uptake in A704 human renal adenocarcinoma cells after 24 h of exposure to concentrations of 1, 2, 3, 4, and 5 µg/mL. Results were normalized to the untreated control, which was considered 100%, and are presented as mean ± standard deviation (SD) of three independent experiments. Statistical significance versus the untreated control was determined separately for each formulation using one-way ANOVA followed by Dunnett’s multiple-comparisons test. Statistical significance is indicated as follows: * *p* < 0.05; ** *p* < 0.01; *** *p* < 0.001; and **** *p* < 0.0001.

Neutral red uptake by A704 cells was reduced following 24 h of exposure to both magnetic colloidal suspensions, with distinct concentration–response profiles for MCS 1 and MCS 2 (Figure 6 and Figure 7). MCS 1 produced a progressive decrease in neutral red uptake across the tested concentration range. Uptake values decreased from 88.46% of the untreated control at 1 µg/mL to 82.91% at 2 µg/mL and 75.33% at 3 µg/mL. A more pronounced reduction was observed at the two highest concentrations, with values of 64.80% at 4 µg/mL and 62.98% at 5 µg/mL. MCS 2 induced comparatively modest reductions at concentrations of 1–3 µg/mL, with neutral red uptake values of 85.68%, 83.01%, and 78.05%, respectively. The response became substantially more pronounced at 4 and 5 µg/mL, where neutral red uptake decreased to 58.89% and 50.04% of the control, respectively. Thus, at the highest tested concentration, MCS 2 reduced neutral red uptake to approximately one-half of the control level.

The microscopic staining pattern was consistent with the quantitative findings, showing a visible reduction in intracellular neutral red staining at increasing concentrations, particularly following treatment with MCS 2 at 4 and 5 µg/mL. Overall, both formulations reduced the capacity of A704 cell cultures to accumulate and retain neutral red, with MCS 2 exerting a numerically stronger effect at the highest concentrations. Because neutral red uptake depends on both the number of viable cells and lysosomal dye accumulation, these findings indicate cytotoxic and lysosome-associated alterations but do not, by themselves, demonstrate selective lysosomal damage.

#### 2.2.4. Mitochondrial Membrane Depolarization Assessed by JC-1

**Figure 8:**
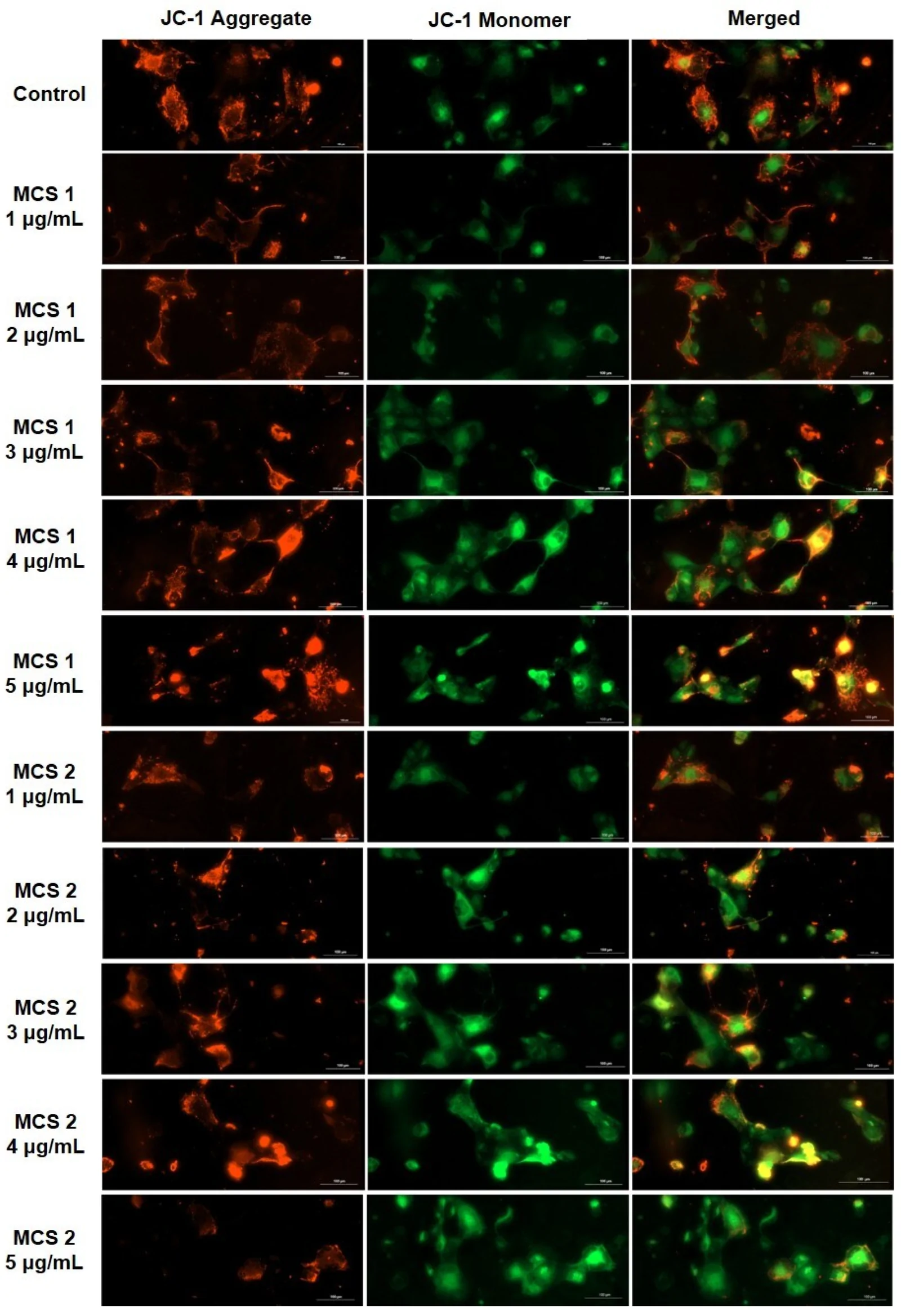
Representative JC-1 fluorescence images of A704 human renal adenocarcinoma cells following 24 h of exposure to MCS 1 and MCS 2 at concentrations of 1, 2, 3, 4, and 5 µg/mL. Red fluorescence corresponds predominantly to JC-1 aggregates associated with polarized mitochondria, whereas green fluorescence corresponds to JC-1 monomers associated with reduced mitochondrial membrane potential. The merged images illustrate the relative distribution of the two fluorescence signals. Images were acquired at 20× magnification. Scale bars: 100 µm.

**Figure 9:**
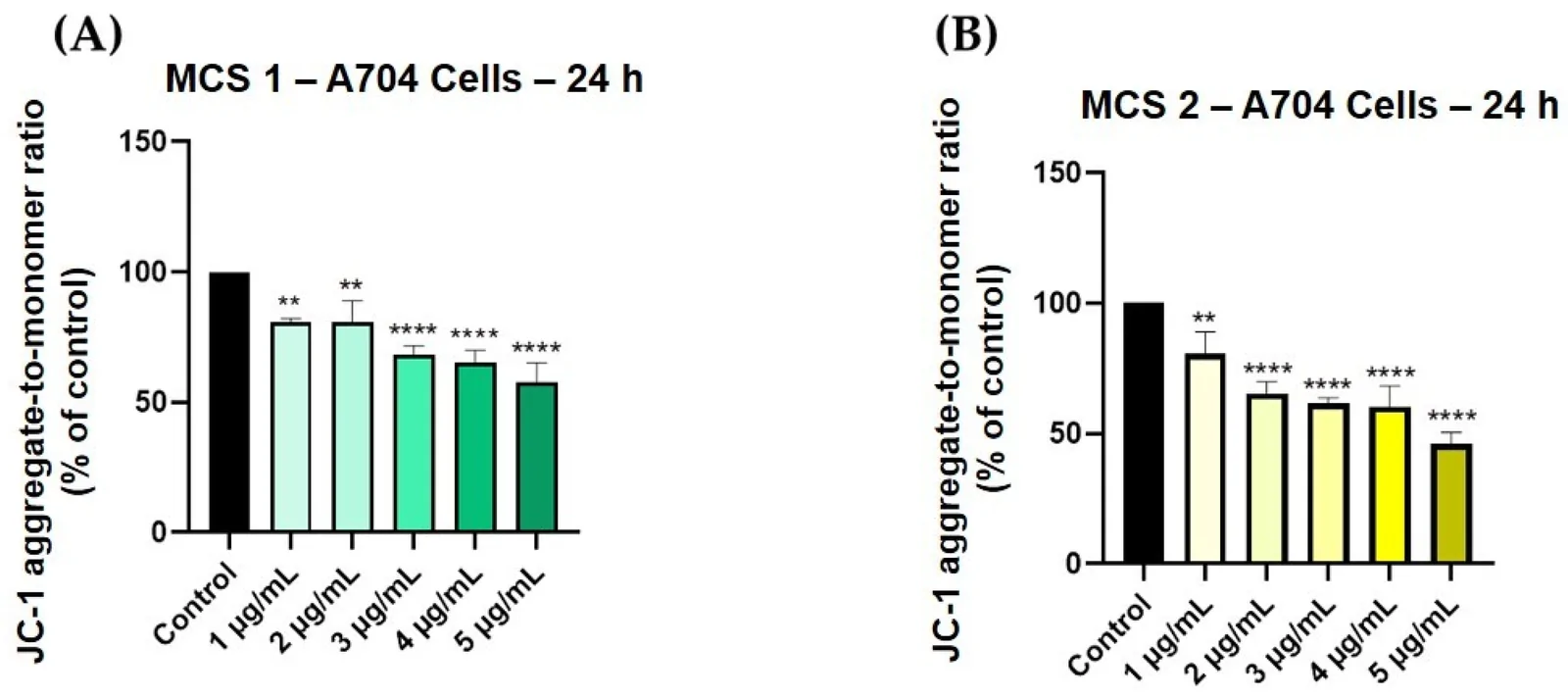
Effects of MCS 1 (**A**) and MCS 2 (**B**) on the JC-1 aggregate-to-monomer fluorescence ratio in A704 human renal adenocarcinoma cells following 24 h of exposure to concentrations of 1, 2, 3, 4, and 5 µg/mL. Results were normalized to the untreated control, which was considered 100%, and are presented as mean ± standard deviation (SD) of three independent experiments. Statistical significance versus the untreated control was determined separately for each formulation using one-way ANOVA followed by Dunnett’s multiple-comparisons test. Statistical significance is indicated as follows: ** *p* < 0.01 and **** *p* < 0.0001.

Exposure of A704 cells to MCS 1 and MCS 2 for 24 h altered the JC-1 fluorescence pattern, indicating a reduction in mitochondrial membrane potential (ΔΨm) (Figure 8 and Figure 9). Untreated cells displayed a predominance of red JC-1 aggregate fluorescence, consistent with preserved mitochondrial polarization. In treated cultures, the red fluorescence progressively decreased, whereas green JC-1 monomer fluorescence became more prominent, particularly at the higher concentrations.

For MCS 1, the JC-1 aggregate-to-monomer fluorescence ratio decreased to 80.65% and 80.56% of the control at 1 and 2 µg/mL, respectively. A more pronounced reduction was observed from 3 µg/mL onward, with values of 68.22% at 3 µg/mL, 65.35% at 4 µg/mL, and 57.69% at 5 µg/mL. Thus, MCS 1 produced a modest reduction at the two lowest concentrations, followed by a greater decline at concentrations of 3–5 µg/mL.

MCS 2 induced a stronger overall reduction in the JC-1 aggregate-to-monomer ratio. The values decreased from 80.56% of the control at 1 µg/mL to 65.35% at 2 µg/mL, 61.56% at 3 µg/mL, and 60.35% at 4 µg/mL. At 5 µg/mL, the ratio reached 46.02% of the control, representing the most pronounced mitochondrial depolarization observed among the tested conditions.

The quantitative findings were consistent with the fluorescence images, which showed an overall shift from red aggregate fluorescence toward green monomer fluorescence. Both formulations therefore altered mitochondrial polarization in A704 cells, with MCS 2 producing numerically greater effects than MCS 1 at concentrations of 2–5 µg/mL. Because the statistical analysis compared each concentration with the corresponding untreated control, differences between MCS 1 and MCS 2 were interpreted descriptively. The decrease in the JC-1 aggregate-to-monomer ratio indicates mitochondrial membrane depolarization but does not, by itself, establish irreversible mitochondrial injury or confirm a specific cell-death mechanism.

#### 2.2.5. Nuclear and Mitochondrial Alterations Assessed by Hoechst 33342 and MitoTracker Red CMXRos

**Figure 10:**
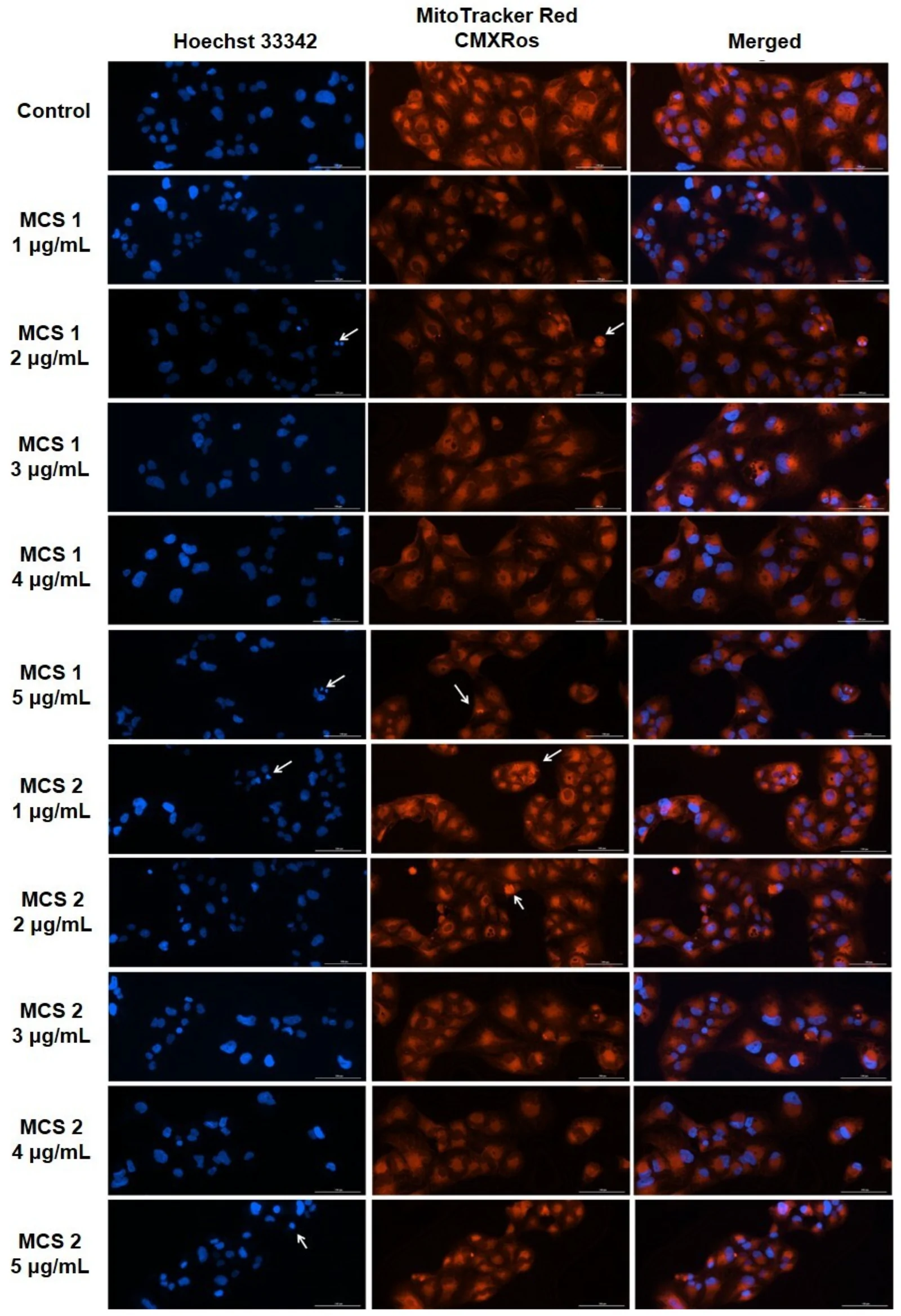
Representative fluorescence images of nuclear morphology and mitochondrial staining patterns in A704 human renal adenocarcinoma cells following 24 h of exposure to MCS 1 and MCS 2 at concentrations of 1, 2, 3, 4, and 5 µg/mL. Nuclei were stained with Hoechst 33342, whereas mitochondria were visualized using MitoTracker Red CMXRos. The merged images show the spatial relationship between the nuclear and mitochondrial fluorescence signals. White arrows indicate representative cells exhibiting altered nuclear morphology or changes in the mitochondrial staining pattern. Images were acquired at 20× magnification. Scale bars: 100 µm.

Qualitative fluorescence microscopy revealed treatment-associated alterations in nuclear morphology and mitochondrial staining patterns following 24 h of exposure to MCS 1 and MCS 2 (Figure 10). Untreated A704 cells displayed predominantly regular, oval nuclei with relatively uniform Hoechst 33342 fluorescence. MitoTracker Red CMXRos staining showed a broadly distributed mitochondrial signal throughout the cytoplasmic compartment. Following exposure to MCS 1, the overall nuclear and mitochondrial appearance remained relatively preserved at the lower concentrations. Nevertheless, a subset of cells exhibited smaller or more intensely stained nuclei, together with localized changes in the distribution of the MitoTracker signal. These alterations became more apparent at the highest tested concentration, where reduced cell density and a less uniform mitochondrial staining pattern were observed.

MCS 2 produced more evident qualitative changes, particularly at the higher concentrations. The treated cultures contained a greater proportion of cells with smaller, irregular, or more intensely stained nuclei, compatible with chromatin condensation. The MitoTracker fluorescence pattern also became less homogeneous and more punctate in several cells, suggesting altered mitochondrial organization and/or reduced probe accumulation. These changes were accompanied by an apparent reduction in the number of adherent cells within the microscopic fields, especially at 4 and 5 µg/mL.

Overall, the qualitative fluorescence findings indicate that both formulations affected nuclear appearance and mitochondrial staining, with more pronounced alterations observed following MCS 2 treatment. These observations are consistent with the reduction in mitochondrial membrane potential detected by the JC-1 assay. However, because MitoTracker Red CMXRos accumulation depends partly on mitochondrial membrane potential, changes in fluorescence intensity or distribution cannot be interpreted exclusively as structural mitochondrial damage. Likewise, the nuclear changes were considered apoptosis-associated morphological features rather than definitive evidence of apoptotic cell death.

#### 2.2.6. Cell-Death-Associated Morphology Assessed by AO/PI Dual Staining

**Figure 11:**
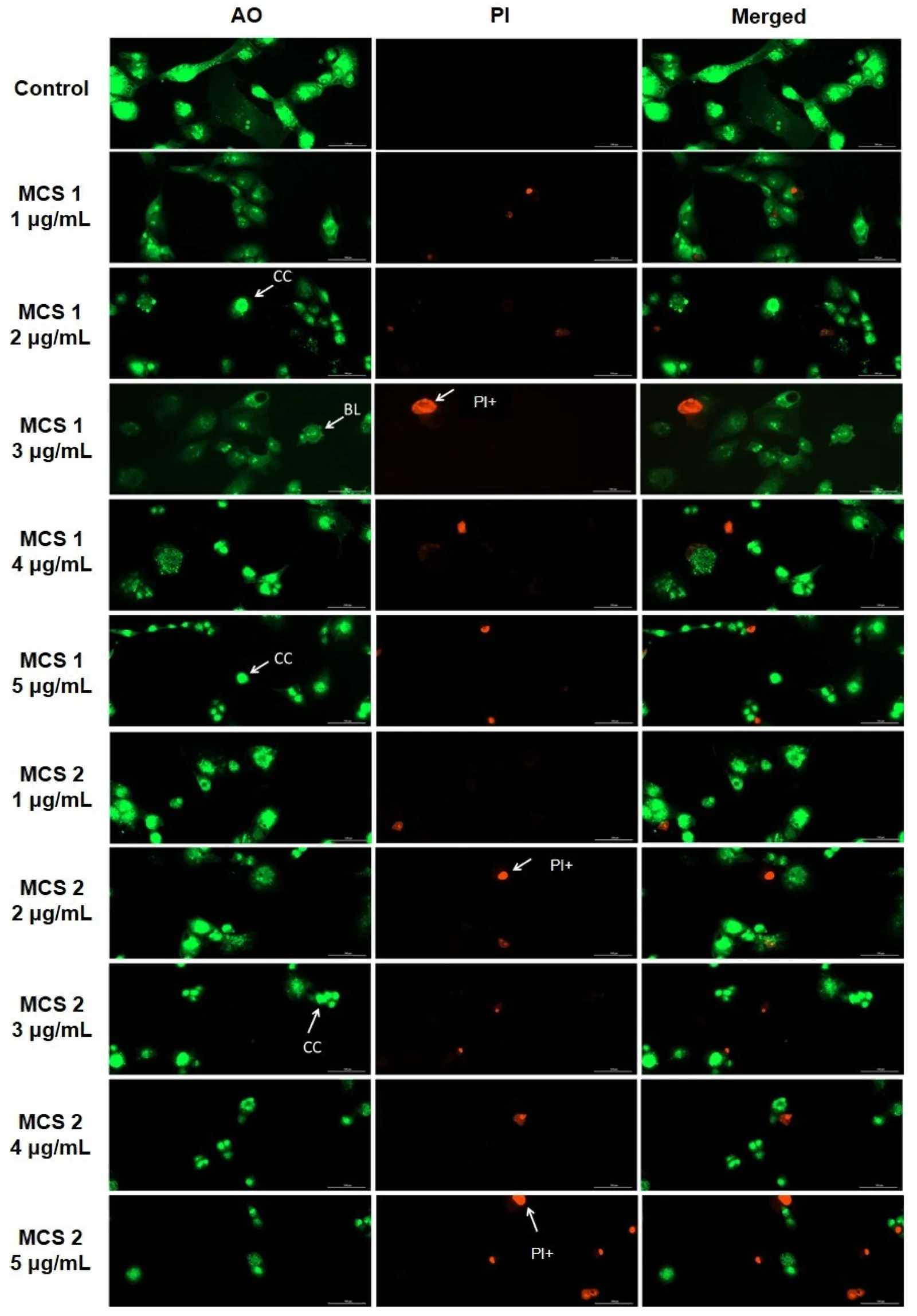
Representative fluorescence images of A704 human renal adenocarcinoma cells following 24 h of exposure to MCS 1 and MCS 2 at concentrations of 1, 2, 3, 4, and 5 µg/mL, assessed by acridine orange/propidium iodide dual staining. Acridine orange fluorescence indicates nucleated cells, whereas propidium iodide uptake identifies cells with compromised plasma membrane integrity. White arrows indicate representative morphological alterations, including chromatin condensation, membrane blebbing, and propidium-iodide-positive cells. Images were acquired at 20× magnification. Scale bars: 100 µm. CC, chromatin condensation; BL, membrane blebbing; PI+, propidium-iodide-positive cell.

Acridine orange/propidium iodide dual staining was used to qualitatively examine treatment-associated changes in nuclear morphology and plasma membrane integrity after 24 h of exposure to MCS 1 and MCS 2 (Figure 11). Untreated A704 cells exhibited predominantly uniform green fluorescence, with regularly shaped nuclei and minimal propidium iodide uptake, consistent with preserved membrane integrity. Following treatment with MCS 1, most cells retained green acridine orange fluorescence; however, selected cells displayed brighter and more condensed nuclear staining, compatible with chromatin condensation. Cell shrinkage and membrane blebbing were also observed in some microscopic fields, particularly at intermediate and higher concentrations. Scattered propidium-iodide-positive cells were detected, indicating loss of plasma membrane integrity in a subpopulation of treated cells.

MCS 2 produced more evident qualitative alterations, including reduced cell density, cell shrinkage, and an increased occurrence of condensed or irregularly shaped nuclei. Propidium-iodide-positive cells were observed at several concentrations and were most apparent following exposure to 5 µg/mL MCS 2. The greater proportion of red- or orange-fluorescent cells at the highest concentration indicated a more pronounced loss of membrane integrity compared with the untreated control.

Overall, both magnetic colloidal suspensions induced morphological features associated with cell death, with more pronounced alterations observed following MCS 2 treatment, particularly at 5 µg/mL. These findings were consistent with the reductions in metabolic activity, neutral red uptake, and mitochondrial membrane potential. Nevertheless, because the AO/PI assessment was qualitative and no quantitative classification of viable, apoptotic, and necrotic cells was performed, the observed fluorescence patterns were interpreted as apoptosis- and membrane-damage-associated features rather than definitive confirmation of a specific cell-death mechanism.

#### 2.2.7. Long-Term Effects on A704 Cell Clonogenic Capacity

**Figure 12:**
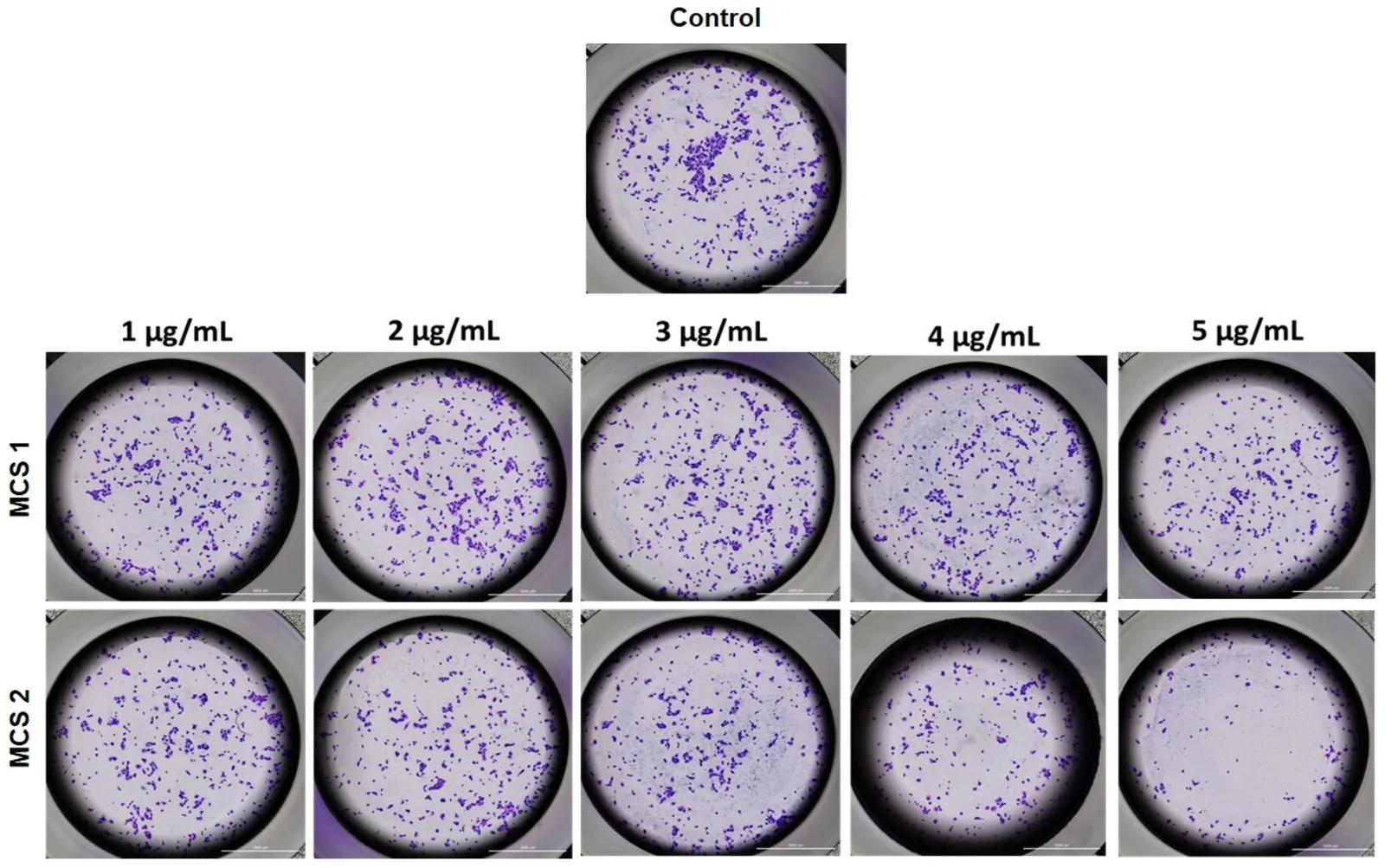
Representative images of crystal-violet-stained colonies formed by A704 human renal adenocarcinoma cells following 24 h of exposure to MCS 1 and MCS 2 at concentrations of 1, 2, 3, 4, and 5 µg/mL, followed by 7 days of colony development in treatment-free medium. Untreated cells served as the control. Scale bar: 2000 µm.

**Figure 13:**
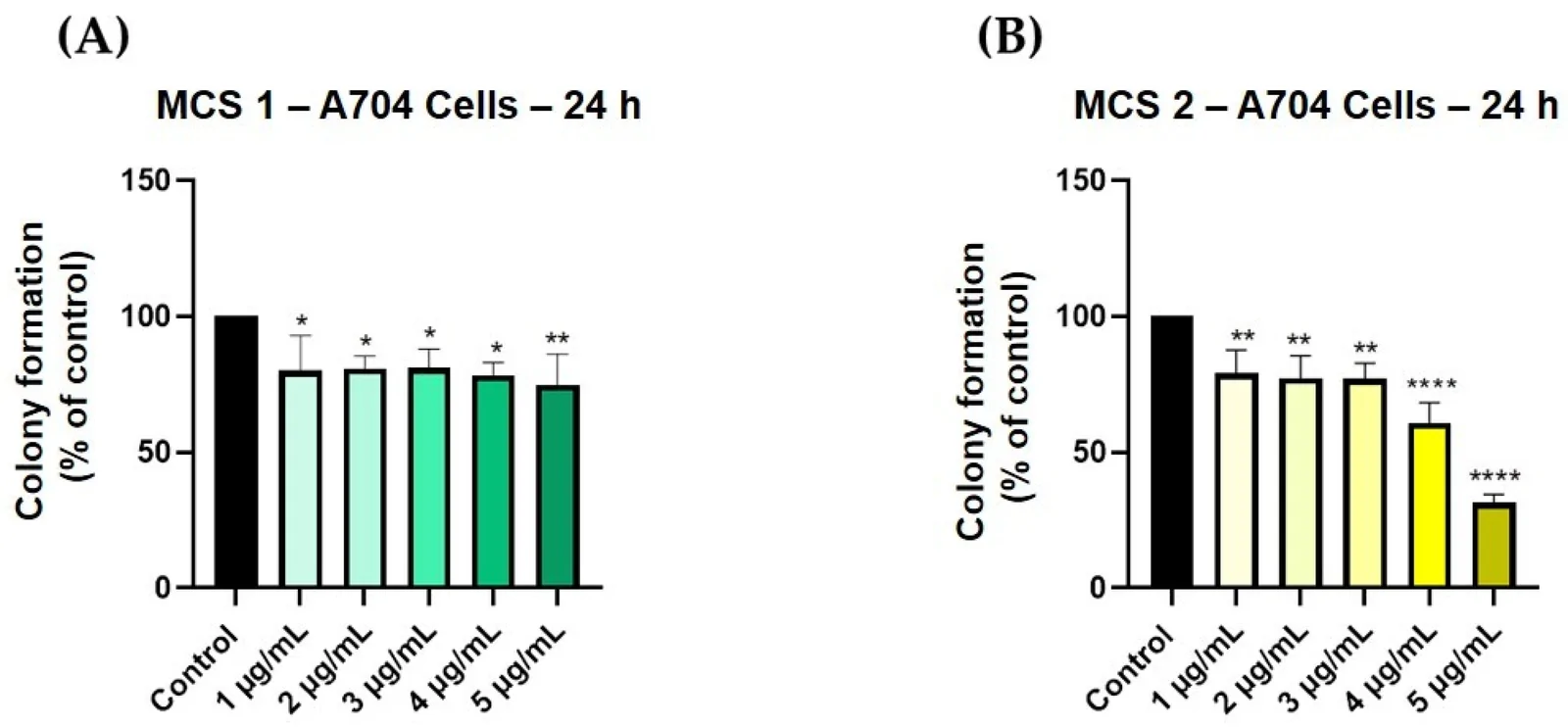
Effects of MCS 1 (**A**) and MCS 2 (**B**) on the clonogenic capacity of A704 human renal adenocarcinoma cells following 24 h of exposure to concentrations of 1, 2, 3, 4, and 5 µg/mL and subsequent colony development for 7 days in treatment-free medium. Results were normalized to the untreated control, which was considered 100%, and are presented as mean ± standard deviation of three independent experiments. Statistical significance versus the untreated control was determined separately for each formulation using one-way ANOVA followed by Dunnett’s multiple-comparisons test. Statistical significance is indicated as follows: * *p* < 0.05; ** *p* < 0.01; and **** *p* < 0.0001.

The long-term effects of MCS 1 and MCS 2 on the clonogenic capacity of A704 cells were evaluated after a 24 h exposure period followed by 7 days of colony development in treatment-free medium (Figure 12 and Figure 13). Both formulations reduced colony formation relative to the untreated control, although distinct response patterns were observed.

MCS 1 produced a modest reduction in clonogenic capacity across the tested concentration range. Colony formation remained at 80.21%, 80.23%, and 80.21% of the control following exposure to 1, 2, and 3 µg/mL, respectively. A slightly greater reduction was observed at 4 and 5 µg/mL, where colony formation decreased to 78.39% and 74.93%, respectively. These findings indicate a relatively stable inhibitory effect of MCS 1, with no clear concentration-dependent response over most of the tested range.

MCS 2 produced a more pronounced concentration-related reduction in colony formation. Clonogenic capacity decreased to 79.22%, 77.35%, and 77.02% of the control at 1, 2, and 3 µg/mL, respectively. A substantially stronger effect was observed at the two highest concentrations, with colony formation decreasing to 60.67% at 4 µg/mL and 31.44% at 5 µg/mL. Thus, exposure to 5 µg/mL MCS 2 resulted in a 68.56% reduction in colony formation relative to the untreated control.

The qualitative appearance of the crystal-violet-stained colonies was consistent with the quantitative findings, showing a visible reduction in colony density, particularly following exposure to MCS 2 at 4 and 5 µg/mL. Overall, MCS 2 exerted numerically stronger long-term antiproliferative effects than MCS 1 at the highest tested concentrations. However, because the statistical analysis compared each treatment group with its corresponding untreated control, differences between the two formulations were interpreted descriptively rather than as direct statistically significant comparisons.

## 3. Discussion

Nanomedicine-based approaches have been investigated for diagnostic and therapeutic applications in oncology, including renal cell carcinoma [16,17].

The two magnetic colloidal suspensions investigated in the present study were prepared from precursor nanopowders with markedly different structural and magnetic characteristics. MIONPs_1 consisted of magnetite (Fe_3_O_4_) with an average crystallite size of 18 nm, a BET-derived particle diameter of 21 nm, and a specific surface area of 56 m^2^/g. In contrast, MIONPs_2 consisted of maghemite (γ-Fe_2_O_3_) with substantially smaller crystallite and BET-derived particle dimensions of 5 and 8 nm, respectively, and a considerably larger specific surface area of 149 m^2^/g [18,19]. The smaller dimensions of MIONPs_2 are therefore consistent with the greater surface area available per unit mass. The two precursors also differed magnetically: MIONPs_1 exhibited higher saturation magnetization, remanent magnetization, and coercivity than MIONPs_2. These differences should not be attributed exclusively to the magnetite or maghemite phase, because the magnetic response of iron oxide nanoparticles is jointly influenced by particle size, crystallinity, internal spin order, surface structure, morphology, and interparticle interactions. Previous experimental studies have shown that decreasing particle dimensions may increase the relative contribution of magnetically disordered surface spins, whereas crystallinity and internal magnetic order can be equally or even more important than nominal particle size in determining saturation magnetization [6,20,21].

At the colloidal-suspension level, MCS 2 exhibited a volumetric saturation magnetization of 1.541 Gs, which was approximately 45.5% higher than the value of 1.059 Gs measured for MCS 1. This result does not contradict the higher mass-specific saturation magnetization previously reported for the uncoated MIONPs_1 powder. The powder values were expressed per unit mass of magnetic material, whereas the magnetization of the colloidal suspensions was measured per unit volume of the entire dispersion and therefore depended strongly on the amount of magnetic solid present in that volume. Accordingly, the VSM-derived magnetic-particle volume fraction and mass concentration were higher for MCS 2 than for MCS 1. These calculated values should be regarded as formulation-level estimates derived from the VSM signal rather than as independent measurements of particle loading. Moreover, the similar magnetic fields required to reach 95% of saturation for MCS 1 and MCS 2 indicate comparable approaches to high-field saturation under the experimental conditions. Because only virgin magnetization curves were recorded for the colloidal suspensions, the present data do not independently establish superparamagnetic behavior or permit determination of the coercivity and remanence of the final formulations.

The double oleic acid coating facilitated the transfer and dispersion of the initially hydrophobic iron oxide nanoparticles in the aqueous phase. Bilayer oleic acid coatings have previously been used to obtain water-dispersible iron oxide nanoparticles, although the resulting colloidal behavior remains dependent on the organization of the surfactant layers, pH, ionic strength, and particle–particle interactions [11,22]. Consequently, the dimensions measured by DLS represent hydrodynamic entities that include the inorganic particle core, the organic coating, the associated solvent layer, and any particle assemblies present in suspension. They are therefore expected to exceed the crystallite and primary particle dimensions determined from XRD, BET analysis, or electron microscopy. In agreement with this interpretation, Soares et al. reported oleic-acid-bilayer-stabilized iron oxide nanoparticles with an approximately 9 nm inorganic core but a hydrodynamic diameter of approximately 170 nm in aqueous dispersion [12].

Both formulations displayed comparable PDI values, indicating a broadly similar overall width of the hydrodynamic size distributions. Nevertheless, their distribution profiles differed meaningfully. MCS 1 had a Z-average diameter of 103.9 nm and contained a secondary volume-weighted population centered at 228.0 nm, whereas MCS 2 had a lower Z-average diameter of 79.65 nm and exhibited a single volume-weighted population. Notably, the predominant volume-weighted populations of the two formulations were located at similar diameters, 62.84 nm for MCS 1 and 65.22 nm for MCS 2. Therefore, the lower Z-average diameter of MCS 2 is best interpreted as reflecting the absence of the secondary population of larger hydrodynamic entities detected in MCS 1, rather than a uniform reduction in the size of every dispersed particle. The larger secondary population in MCS 1 may represent a minor fraction of particle aggregates or more complex oleic-acid-coated assemblies.

The BF-STEM observations supported the differences detected by DLS and were also consistent with the characteristics of the precursor powders. MCS 1 contained relatively larger quasi-spherical to slightly polyhedral particles forming compact and irregular associations, whereas MCS 2 displayed apparently finer particles distributed within more diffuse assemblies. The finer morphology of MCS 2 is consistent with the smaller crystallite and BET-derived dimensions of its MIONPs_2 precursor. However, because BF-STEM analysis was performed after deposition and drying of the samples on microscopy grids, the observed particulate associations should be regarded as dry-state morphology and not as direct evidence of the aggregation state present in the aqueous suspension or in the cell-culture medium. EDX mapping confirmed the spatial association of Fe and O in both formulations, supporting the iron oxide nature of the dispersed particles. The high carbon signal should not be used to compare the density of the oleic acid coating between the two samples, because it may originate from both the organic coating and the carbonaceous microscopy support. Likewise, the locally determined elemental weight percentages should not be interpreted as bulk compositional differences between MCS 1 and MCS 2.

Taken together, the physicochemical findings indicate that MCS 1 and MCS 2 represent distinct nanoparticle systems rather than equivalent suspensions differing only in iron oxide phase. Compared with MCS 1, MCS 2 combined a smaller Z-average hydrodynamic diameter, the absence of a detectable secondary population of larger hydrodynamic entities, finer primary particles, and a precursor powder with a substantially higher specific surface area. Particle size, agglomeration state, and interfacial properties have been shown to influence the cellular association and uptake of magnetic iron oxide nanoparticles [10]. These characteristics may therefore have contributed to the more pronounced biological effects subsequently observed for MCS 2. Nevertheless, cellular uptake, intracellular iron accumulation, and nanoparticle dissolution were not directly measured in the present study; consequently, the relationship between the physicochemical differences and biological potency should be regarded as a mechanistic hypothesis. Furthermore, because the working concentrations used in the biological assays were prepared on the basis of the calculated mass of magnetic phase, the higher stock concentration of MCS 2 should not be invoked as a direct explanation for its stronger effects at the same nominal concentration.

Following physicochemical characterization, the biological effects of MCS 1 and MCS 2 were investigated in A704 human renal adenocarcinoma cells using complementary short- and long-term endpoints. Magnetic nanoparticle-based approaches previously explored in renal cancer have primarily involved drug-loaded magnetic carriers or magnetically actuated particles designed to enhance tumor-cell targeting or induce cell death [9,15]. In contrast, the present study examined the direct effects of unloaded, double-oleic-acid-coated magnetic colloidal suspensions in the absence of an externally applied magnetic field. Both formulations reduced cellular metabolic activity and neutral red uptake, altered cell morphology and mitochondrial polarization, induced cell-death-associated morphological changes, and diminished long-term clonogenic capacity. Across these complementary endpoints, MCS 2 generally produced numerically more pronounced effects than MCS 1 at the highest tested concentrations. However, because the statistical analysis compared each formulation separately with its corresponding untreated control, the difference in potency between MCS 1 and MCS 2 should be interpreted descriptively rather than as a directly demonstrated statistically significant difference.

The MTT assay demonstrated that both formulations significantly reduced the metabolic activity of A704 cells after 24 h of exposure. MCS 1 produced an initial reduction followed by an apparent plateau at concentrations of 2–5 µg/mL, whereas MCS 2 generated a more pronounced concentration-related response, particularly at 4 and 5 µg/mL. Because MTT reduction reflects the overall metabolic capacity of the cell population, these findings should not be interpreted independently as a direct measurement of cell number or as definitive evidence of a specific cell-death mechanism [23].

The bright-field observations supported the quantitative MTT findings. MCS 1 caused a modest reduction in cell density and confluence without marked morphological disruption, whereas MCS 2 produced more evident alterations at 4 and 5 µg/mL. The comparatively greater activity of MCS 2 may be related, at least in part, to its smaller Z-average hydrodynamic diameter, more uniform volume-weighted distribution, and finer primary particles. Previous studies have demonstrated that the size, coating, surface properties, and agglomeration state of iron oxide nanoparticles can substantially modify their cellular association, internalization, intracellular distribution, and cytotoxicity [7,10]. Nevertheless, nanoparticle uptake and intracellular iron accumulation were not quantified in the present investigation, and this physicochemical–biological relationship therefore remains a mechanistic hypothesis.

Neutral red uptake provided a complementary assessment of the response of A704 cells to the two formulations. Both MCS 1 and MCS 2 reduced neutral red uptake in A704 cells, with the greatest reduction observed at 5 µg/mL, where values reached 62.98% and 50.04% of the control, respectively. Because NRU reflects both viable cell number and the capacity of cells to accumulate and retain neutral red within acidic lysosomal compartments [24], the observed decrease cannot be attributed specifically to lysosomal injury. This interpretation is biologically plausible because internalized iron oxide nanoparticles are frequently trafficked through endosomal and lysosomal compartments [7]. Moreover, lysosomal function and autophagic flux are increasingly recognized as relevant determinants of renal carcinoma biology and treatment response. For example, lysosomal sequestration has been implicated in sunitinib resistance in renal clear cell carcinoma, while lysosome-targeting agents have revealed specific vulnerabilities in von Hippel–Lindau (VHL)-inactivated renal carcinoma models [25,26]. Nevertheless, the NRU findings indicate cytotoxic and lysosome-associated alterations rather than direct lysosomal damage, which was not specifically assessed.

Assessment of mitochondrial membrane potential provided additional information regarding the cellular effects induced by the two formulations. JC-1 analysis showed a decrease in the aggregate-to-monomer fluorescence ratio, reaching 57.69% and 46.02% of the control at 5 µg/mL for MCS 1 and MCS 2, respectively. The observed shift from red aggregate to green monomer fluorescence further supported mitochondrial membrane depolarization. Although loss of ΔΨm indicates mitochondrial dysfunction and may accompany intrinsic apoptosis, it is not specific to apoptotic cell death [27].

Mitochondrial involvement in iron oxide nanoparticle-induced cytotoxicity has been demonstrated in other experimental models. Kai et al. reported that Fe_3_O_4_ and oleic-acid-coated Fe_3_O_4_ nanoparticles induced mitochondrial membrane depolarization in BEL-7402 hepatoma cells, together with Bax upregulation, cytochrome c release, and caspase-3 activation [13]. Similarly, Ruan et al. observed marked JC-1 depolarization and cytotoxicity after the mitochondrial accumulation of targeted iron oxide nanoparticles in MCF-7 cells [14]. These studies provide mechanistic precedent for a mitochondrial contribution to iron oxide nanoparticle cytotoxicity, although their particle characteristics, concentrations, targeting strategies, and cellular models differ from those used in the present study.

The Hoechst 33342 and MitoTracker Red CMXRos staining revealed nuclear and mitochondrial alterations, more evident after MCS 2 exposure and consistent with the JC-1 findings. However, as MitoTracker Red CMXRos fluorescence is membrane potential-dependent, these changes should not be interpreted independently as definitive evidence of structural mitochondrial damage [28]. Because reactive oxygen species generation, cytochrome c release, Bcl-2 family proteins, and caspase activation were not evaluated, the combined JC-1, Hoechst, and MitoTracker findings support mitochondrial dysfunction and apoptosis-associated morphology but do not establish activation of the intrinsic apoptotic pathway.

AO/PI dual staining further supported the presence of treatment-associated cell-death morphology and loss of plasma membrane integrity. Both formulations induced morphological alterations and PI-positive cells, with more pronounced effects following exposure to 5 µg/mL MCS 2, consistent with the MTT, NRU, and JC-1 findings.

Nevertheless, the AO/PI evaluation in the present study was qualitative, and PI positivity may reflect loss of membrane integrity occurring in both necrosis and late apoptosis [29,30]. In the absence of additional apoptosis markers, these findings indicate apoptosis-associated morphological changes and membrane compromise rather than definitive apoptosis or necrosis. In the context of assessing the cytotoxicity of iron-based nanoparticles, Kanagesan et al. reported a concentration-dependent increase in cells displaying apoptotic and necrotic features, accompanied by decreased viability, in 4T1 murine breast cancer cells exposed to MnFe_2_O_4_ nanoparticles, as assessed by AO/PI staining [31].

The colony formation assay extended the biological evaluation beyond the immediate 24 h response by determining whether exposed cells retained their long-term reproductive capacity after treatment removal [32].

MCS 1 produced a modest reduction in clonogenic capacity, whereas MCS 2 exerted a more pronounced concentration-dependent effect. At 5 µg/mL, colony formation remained at 74.93% of the control following MCS 1 treatment, compared with 31.44% following MCS 2 treatment. Because colony development occurred for 7 days in treatment-free medium, the marked reduction observed after MCS 2 exposure indicates that a transient 24 h treatment produced a persistent impairment of the capacity of A704 cells to resume sustained proliferation. This finding complements the short-term metabolic and mitochondrial endpoints and suggests that the surviving cell population did not fully recover its clonogenic potential. However, because quantification was based on solubilized crystal violet absorbance, the results represent relative colony-associated biomass and clonogenic capacity rather than a direct manual count of individual colonies.

Taken together, the concordance among metabolic, lysosomal, morphological, mitochondrial, membrane-integrity, and clonogenic endpoints supports a genuine cytotoxic and antiproliferative response of A704 cells to both magnetic colloidal suspensions. MCS 2 consistently produced numerically greater effects at the highest concentrations, particularly in terms of metabolic activity, neutral red uptake, mitochondrial depolarization, membrane-compromise-associated fluorescence, and long-term clonogenic capacity. This response may be related to the distinct physicochemical profile of MCS 2, including its smaller Z-average hydrodynamic diameter, the absence of a detectable secondary population of larger hydrodynamic entities, finer primary particles, and the substantially larger specific surface area of its precursor nanopowder. These properties may increase the accessible particle surface and modify cellular association or intracellular processing. However, the stronger activity of MCS 2 cannot be attributed to a single property, including the γ-Fe_2_O_3_ phase, because particle size, aggregation, coating organization, protein adsorption, cellular uptake, intracellular dissolution, and iron-mediated redox processes may act simultaneously. Moreover, because the biological working solutions were prepared according to the calculated mass of magnetic phase, the higher concentration of the MCS 2 stock suspension does not, by itself, explain its stronger effects at the same nominal treatment concentration.

Several limitations should be considered when interpreting these findings. The experiments were performed only in a single renal carcinoma cell line using a two-dimensional culture model, one exposure duration, and a relatively narrow concentration range. Although the present study was designed to comparatively assess the biological responses of A704 cells to the two magnetic colloidal suspensions, a non-malignant human renal epithelial comparator was not included. Moreover, the effects of the formulations on healthy cells of other tissue origins were not investigated. Therefore, tumor selectivity, a potential therapeutic window, and the broader biosafety profiles of the formulations cannot yet be established. In addition, cellular nanoparticle uptake, intracellular iron concentration, reactive oxygen species generation, lysosomal membrane integrity, autophagic flux, Annexin V positivity, caspase activation, and cytochrome c release were not determined. Engineered nanoparticles may also interfere with optical or colorimetric assays in a formulation-dependent manner; although the treatment medium was removed before MTT analysis and the agreement among several independent endpoints strengthens the biological interpretation, future experiments should include formulation-specific cell-free interference controls [33]. Further studies should therefore extend the evaluation to additional renal cancer cell lines and more complex cellular models, including three-dimensional systems, such as spheroids, organoids, and tissue-based models, together with non-malignant renal cells and healthy cell models of different tissue origins. These investigations will be important for providing a more comprehensive assessment of the biological effects and for defining the selectivity and safety profile of the formulations. In addition, future studies should characterize colloidal behavior in complete culture medium, quantify cellular uptake and intracellular iron, and investigate oxidative, lysosomal, and mitochondrial cell-death pathways using quantitative molecular and flow-cytometric methods. In vivo investigations will also be necessary before the therapeutic relevance and biosafety of either formulation can be established.

## 4. Materials and Methods

### 4.1. Synthesis and Characterization of Magnetic Colloidal Suspensions

Two previously developed magnetic iron oxide nanopowders (MIONPs) were used as precursor materials for the preparation of the magnetic colloidal suspensions (MCSs) investigated in the present study. The first precursor, hereinafter designated MIONPs_1, consisted of magnetite (Fe_3_O_4_) nanoparticles obtained by solution combustion synthesis using iron(III) nitrate nonahydrate [Fe(NO_3_)_3_·9H_2_O] as the oxidizing agent and citric acid monohydrate (C_6_H_8_O_7_·H_2_O) as the fuel. The combustion reaction was performed under an oxygen-restricted atmosphere, as previously described by Ianoş et al. [18]. The second precursor, designated MIONPs_2, consisted of maghemite (γ-Fe_2_O_3_) nanoparticles prepared by solution combustion synthesis using Fe(NO_3_)_3_·9H_2_O as the oxidizing agent and D-(+)-glucose (C_6_H_12_O_6_) as the fuel. Following combustion synthesis, the resulting powder was chemically treated with hydrogen peroxide (H_2_O_2_) to oxidize and remove residual carbon from the nanoparticle surface, according to the procedure previously reported by Ianoş et al. [19]. The principal physicochemical characteristics previously reported for the two precursor nanopowders are summarized in Table 2.

Following the preparation of the two MIONP precursor powders, MCS 1 and MCS 2 were obtained according to previously published protocols developed by our research group [34,35], with minor adaptations. Briefly, 1 g of each MIONP sample was dispersed in a mixture containing 10 mL of 96% ethanol and 200 mL of distilled water and maintained for 24 h. Each mixture was subsequently sonicated for 2 h at 50% amplitude using a Q700 sonicator, with alternating cycles of 10 s pulse on and 5 s pulse off. The resulting dispersions were subjected to thermomagnetic stirring, and the first oleic acid layer was added when the temperature reached 80–82 °C. The single-coated MIONPs were washed three times with distilled water. The pH was then adjusted from neutral to alkaline using 25% ammonium hydroxide solution, after which the second oleic acid layer was added at 80–82 °C. Two aqueous magnetic colloidal suspensions containing double oleic-acid-coated MIONPs were thus obtained and subsequently designated MCS 1, prepared from MIONPs_1, and MCS 2, prepared from MIONPs_2.

Both MCSs were initially characterized in terms of saturation magnetization using a VSM 880 vibrating-sample magnetometer (DMS/ADE Technologies, Westwood, MA, USA). Based on the saturation magnetization values, the volume fraction of the dispersed magnetic phase, *φ*, was estimated according to the following relationship [36]:

$$\varphi =\frac{M_{s}}{M_{d}}$$

where *M_s_* is the saturation magnetization of the magnetic colloidal suspension, expressed in Gs, and *M_d_* is the saturation magnetization per unit volume of the dispersed magnetic solid phase, also expressed in Gs. Based on previously reported calibration data for water-based magnetic fluids, according to which a magnetic-particle volume fraction of 0.07 corresponds to a saturation magnetization of approximately 300 Gs, an effective Md value of approximately 4.29 × 10^3^ Gs was used in the calculation [37].

The mass concentration of the dispersed magnetic phase was subsequently calculated using the following relationship:

$$C_{m}=\;\varphi \cdot \rho$$

where *C_m_* represents the mass concentration of the dispersed magnetic phase and *ρ* is the theoretical density of the corresponding iron oxide. A density of 5.2 g/cm^3^, corresponding to magnetite (Fe_3_O_4_), was used for MCS 1, whereas the theoretical density of maghemite (γ-Fe_2_O_3_), 4.89 g/cm^3^, was used for MCS 2. The resulting concentrations were expressed as milligrams of Fe_3_O_4_ per milliliter of MCS 1 and milligrams of γ-Fe_2_O_3_ per milliliter of MCS 2 and were subsequently used to prepare the working concentrations employed in the biological assays.

The hydrodynamic size distribution of MCS 1 and MCS 2 was determined by dynamic light scattering (DLS) using a Zetasizer Nano ZS instrument (Malvern Instruments Ltd., Malvern, Worcestershire, UK).

The morphology and ultrastructural characteristics of the magnetic nanoparticles dispersed within MCS 1 and MCS 2 were examined by bright-field scanning transmission electron microscopy (BF-STEM) using an HD-2300 scanning transmission electron microscope (Hitachi High-Technologies Corporation, Tokyo, Japan) operated at an accelerating voltage of 200 kV. Representative BF-STEM images were acquired using the transmitted-electron detector at a magnification of ×300,000. The elemental composition and spatial distribution of carbon, iron, and oxygen within selected nanoparticle-containing regions were investigated by energy-dispersive X-ray spectroscopy (EDX) coupled to the STEM instrument.

### 4.2. In Vitro Biological Assessment

#### 4.2.1. Reagents and Equipment Used for In Vitro Experiments

Cell culture reagents, including trypsin–ethylenediaminetetraacetic acid (trypsin–EDTA) solution, phosphate-buffered saline (PBS), Eagle’s Minimum Essential Medium (EMEM), penicillin–streptomycin solution, and fetal bovine serum (FBS), were obtained from the American Type Culture Collection (ATCC, Manassas, VA, USA). The Cell Proliferation Kit I (MTT), containing the MTT labeling reagent and the corresponding solubilization solution, was purchased from Roche Diagnostics GmbH (Vienna, Austria). Hoechst 33342 and MitoTracker Red CMXRos, used for nuclear and mitochondrial staining, respectively, were purchased from Thermo Fisher Scientific (Waltham, MA, USA). The JC-1 Mitochondrial Membrane Potential Assay Kit was obtained from Elabscience (Houston, TX, USA). Acridine orange (AO), propidium iodide (PI), and sodium lauryl sulfate (SLS) were purchased from Sigma-Aldrich, Merck KGaA (Darmstadt, Germany). Paraformaldehyde solution, 4% in PBS, was obtained from Santa Cruz Biotechnology (Dallas, TX, USA), whereas the 1% crystal violet stock solution was purchased from Electron Microscopy Sciences (Hatfield, PA, USA).

Absorbance and fluorescence measurements were performed using a Cytation 5 multimode plate reader, whereas bright-field and fluorescence images were acquired using a Lionheart FX automated microscope. Both instruments were manufactured by BioTek Instruments, Inc. (Winooski, VT, USA). Data acquisition and analysis were performed using Gen5 Microplate Data Collection and Analysis Software, version 3.14 (BioTek Instruments, Inc., Winooski, VT, USA).

#### 4.2.2. Cell Culture

The A704 human renal adenocarcinoma cell line (ATCC HTB-45^TM^) was obtained from the American Type Culture Collection (ATCC, Manassas, VA, USA). Cells were cultured in Eagle’s Minimum Essential Medium (EMEM; ATCC, Catalog No. 30-2003) supplemented with 10% fetal bovine serum and 1% penicillin–streptomycin solution, corresponding to final concentrations of 100 U/mL penicillin and 100 µg/mL streptomycin. Cell cultures were maintained at 37 °C in a humidified atmosphere containing 5% CO_2_. Cells were routinely monitored by light microscopy and were subcultured upon reaching approximately 80–90% confluence using trypsin–EDTA solution. Cells from early passages were used throughout the experiments.

#### 4.2.3. Experimental Treatment Conditions

For the short-term biological assays, A704 cells were seeded in 96-well plates at a density of 1 × 10^4^ cells/well in complete culture medium and allowed to reach approximately 70% confluence before treatment. Cells were subsequently exposed for 24 h to MCS 1 or MCS 2 at concentrations of 1, 2, 3, 4, and 5 µg/mL. For the colony formation assay, cells were seeded in 96-well plates at a density of 100 cells/well, allowed to adhere for 2 h, and subsequently exposed to MCS 1 or MCS 2 using the same concentrations and exposure duration described above. For each assay, untreated cells maintained under the corresponding experimental conditions served as the negative control.

#### 4.2.4. Assessment of Cell Viability Using the MTT Assay

The effects of MCS 1 and MCS 2 on the metabolic activity and viability of A704 cells were evaluated using the MTT assay, according to previously described methods [38,39], with minor adaptations. Following the 24 h exposure period, the treatment medium was removed and replaced with 100 µL of fresh culture medium. Subsequently, 10 µL of MTT labeling reagent was added to each well, and the plates were incubated for 3 h at 37 °C in a humidified atmosphere containing 5% CO_2_. Thereafter, 100 µL of solubilization solution was added to each well, and the plates were maintained at room temperature for 30 min to ensure the dissolution of the formazan crystals. Absorbance was measured at 570 nm using 630 nm as the reference wavelength with a Cytation 5 multimode plate reader. Cell viability was expressed as a percentage relative to the untreated control, which was considered 100% viable.

Cell viability was calculated using the following equation:

$$Cell\;viability\;\left(\%\right)=\frac{A_{treated}}{A_{control}}\times 100$$

where *A_treated_*—represents the absorbance of cells exposed to MCS 1 or MCS 2, and *A_control_*—represents the absorbance of untreated cells.

#### 4.2.5. Bright-Field Morphological Examination

To evaluate treatment-induced changes in A704 cell morphology, at the end of the exposure period, cellular morphology and confluence were examined under bright-field illumination using a Lionheart FX automated microscope (BioTek Instruments, Inc., Winooski, VT, USA). Representative images were acquired at 20× magnification and processed using Gen5 Microplate Data Collection and Analysis Software, version 3.14 (BioTek Instruments, Inc., Winooski, VT, USA).

#### 4.2.6. Neutral Red Uptake Assay

The cytotoxic effects of MCS 1 and MCS 2 on A704 cells were further evaluated using the neutral red uptake (NRU) assay, which assesses cell viability based on the ability of viable cells to incorporate and retain the neutral red dye within lysosomes [40]. Following the exposure period, the treatment medium was removed and replaced with 100 µL/well of neutral red working solution prepared in EMEM at a final concentration of 40 µg/mL. The plates were incubated for 2 h at 37 °C in a humidified atmosphere containing 5% CO_2_ to allow lysosomal uptake of the dye. The neutral red solution was then removed, and the cells were gently washed with 150 µL/well of PBS to eliminate the excess extracellular dye. Bright-field images were acquired using a Lionheart FX automated microscope before dye extraction.

Subsequently, 150 µL/well of destaining solution, consisting of 50% ethanol, 49% ultrapure water, and 1% glacial acetic acid, was added to extract the intracellular neutral red dye. The absorbance was measured at 540 nm using a Cytation 5 multimode plate reader. Neutral red uptake was expressed as a percentage relative to the untreated control, which was considered 100%. Neutral red uptake was calculated according to the following equation:

$$NRU\;\left(\%\right)=\;\frac{A_{treated}}{A_{control}}\times 100$$

where *A_treated_*—represents the absorbance of cells exposed to MCS 1 or MCS 2, and *A_control_*—represents the absorbance of untreated cells.

#### 4.2.7. Assessment of Mitochondrial Membrane Potential Using the JC-1 Assay

The effects of MCS 1 and MCS 2 on the mitochondrial membrane potential (ΔΨm) of A704 cells were evaluated using the fluorescent JC-1 assay, according to a previously described protocol [41], with minor adaptations. For this assay, cells were seeded in black-walled, clear-bottom 96-well plates. Following the exposure period, the treatment medium was removed, and the cells were washed with PBS. The cells were then incubated with 100 µL/well of JC-1 working solution at a final concentration of 5 µM for 45 min at 37 °C in a humidified atmosphere containing 5% CO_2_, protected from light. After incubation, the staining solution was removed, and the cells were washed twice with PBS to eliminate excess dye.

Fluorescence images were acquired using a Lionheart FX automated microscope and processed using Gen5 Microplate Data Collection and Analysis Software, version 3.14. Fluorescence intensity corresponding to JC-1 aggregates and JC-1 monomers was subsequently measured using a Cytation 5 multimode plate reader. The mitochondrial membrane potential was evaluated by calculating the ratio of JC-1 aggregate fluorescence to JC-1 monomer fluorescence. The resulting aggregate-to-monomer ratios were expressed as percentages relative to the untreated control, which was considered 100%.

The JC-1 aggregate-to-monomer fluorescence ratio was calculated using the following equation:

$$JC-1\;ratio=\;\frac{F_{aggregates}}{F_{monomers}}$$

The relative mitochondrial membrane potential was subsequently determined by normalizing the aggregate-to-monomer ratio of each treated group to that of the untreated control:

$$Relative\;∆\psi_{m}\;\left(\%\right)=\;\frac{R_{treated}}{R_{control}}\times 100$$

*F_aggregates_* and *F_monomers_*—represent the fluorescence intensities of JC-1 aggregates and monomers, respectively, whereas *R_treated_* and *R_control_*—represent the corresponding aggregate-to-monomer ratios in treated and untreated cells.

#### 4.2.8. Assessment of Mitochondrial and Nuclear Morphology Using MitoTracker Red CMXRos and Hoechst 33342

The effects of MCS 1 and MCS 2 on mitochondrial and nuclear morphology were examined by fluorescence microscopy using MitoTracker Red CMXRos and Hoechst 33342, respectively, according to a previously described protocol [35], with minor adaptations. Following the exposure period, the treatment medium was removed, and the cells were incubated with MitoTracker Red CMXRos working solution for 30 min at 37 °C in a humidified atmosphere containing 5% CO_2_, protected from light. A 1 mM stock solution of MitoTracker Red CMXRos was prepared in dimethyl sulfoxide (DMSO) and further diluted in complete EMEM. After staining, the cells were washed with complete culture medium to remove excess fluorescent probe. The cells were then fixed with 4% paraformaldehyde in PBS and subsequently washed with PBS. For nuclear staining, Hoechst 33342 diluted 1:2000 in PBS was added to the cells and incubated for approximately 5–10 min at room temperature, protected from light. The staining solution was then removed, and the cells were washed three times with PBS.

Fluorescence images were acquired at 20× magnification using a Lionheart FX automated microscope. Images corresponding to the Hoechst 33342 and MitoTracker Red CMXRos fluorescence channels, together with the corresponding merged images, were acquired and processed using Gen5 Microplate Data Collection and Analysis Software, version 3.14 (BioTek Instruments, Inc., Winooski, VT, USA).

#### 4.2.9. Assessment of Cell-Death-Associated Morphology Using Acridine Orange/Propidium Iodide Dual Staining

The effects of MCS 1 and MCS 2 on A704 cell membrane integrity and cell-death-associated morphology were qualitatively evaluated using acridine orange/propidium iodide (AO/PI) dual staining, according to a previously described protocol [42], with minor adaptations. Thus, after exposure period, fluorescence images were subsequently acquired at a magnification of 20× using a Lionheart FX automated microscope and processed using Gen5 Microplate Data Collection and Analysis Software, version 3.14 (BioTek Instruments, Inc., Winooski, VT, USA).

#### 4.2.10. Colony Formation Assay

The long-term effects of MCS 1 and MCS 2 on the clonogenic capacity of A704 cells were evaluated using a colony formation assay, according to a previously described method [41], with minor adaptations. Following the 24 h exposure period, the treatment medium was removed and replaced with fresh complete culture medium. The cells were then maintained for an additional 7 days under standard culture conditions, with the culture medium renewed periodically during the incubation period. Colony development was monitored by bright-field microscopy. At the end of the incubation period, the culture medium was removed, and the cells were gently washed with PBS. The colonies were fixed with 4% paraformaldehyde in PBS for 10 min at room temperature and subsequently washed with PBS. The fixed colonies were stained with 0.2% crystal violet solution prepared in PBS for 10 min at room temperature. Excess stain was removed by washing the wells twice with distilled water, and the plates were allowed to dry. Representative images of the colonies were acquired using a Lionheart FX automated microscope at 20× magnification.

For quantitative analysis, the crystal violet retained by the colonies was solubilized using 1% sodium lauryl sulfate solution. Absorbance was measured at 550 nm using a Cytation 5 multimode plate reader. Colony formation capacity was expressed as a percentage relative to the untreated control, which was considered 100%.

The relative colony formation was calculated with the following equation:

$$Relative\;colony\;formation\;\left(\%\right)=\;\frac{A_{treated}}{A_{control}}\times 100$$

where *A_treated_*—represents the absorbance measured in wells exposed to MCS 1 or MCS 2, and *A_control_*—represents the absorbance measured in untreated control wells.

The colony formation inhibition was calculated with the following equation:

$$Colony\;formation\;inhibition\;\left(\%\right)=100-relative\;colony\;formation\;(\%)$$

#### 4.2.11. Statistical Analysis

Quantitative assays, including the MTT, neutral red uptake, JC-1, and colony formation assays, were performed in three independent experiments, each including three technical replicates per experimental condition. Data are presented as the mean ± standard deviation (SD).

Statistical analyses were performed using GraphPad Prism software, version 10.2.3 (GraphPad Software, Boston, MA, USA). For each magnetic colloidal suspension, differences between the untreated control and the tested concentrations were evaluated using one-way analysis of variance (ANOVA), followed by Dunnett’s multiple-comparisons test. Results were normalized to the untreated control, which was considered 100%. A *p* value < 0.05 was considered statistically significant. Statistical significance was indicated as follows: * *p* < 0.05; ** *p* < 0.01; *** *p* < 0.001; and **** *p* < 0.0001.

## 5. Conclusions

The present study demonstrates that two double-oleic-acid-coated magnetic colloidal suspensions derived from structurally distinct magnetite and maghemite precursor nanopowders exhibit distinct physicochemical and biological profiles. MCS 2 displayed a comparatively more uniform hydrodynamic profile and produced more pronounced cytotoxic and persistent antiproliferative effects in A704 human renal adenocarcinoma cells, particularly at higher concentrations, whereas MCS 1 elicited a more moderate response.

The concordance among the short- and long-term biological endpoints supports a formulation-dependent cellular response and indicates that the effects extend beyond transient metabolic impairment. Although the physicochemical differences between the two formulations may contribute to their distinct biological activity, a causal relationship with any individual property cannot be established from the present data. Mitochondrial membrane depolarization and cell-death-associated morphological changes suggest the involvement of mitochondrial dysfunction without establishing a specific cell-death pathway. Further mechanistic and comparative studies are required to clarify cellular uptake, selectivity, and biosafety.

## Figures and Tables

**Table 1:** Semi-quantitative EDX elemental composition, expressed as weight percentages, of the selected regions analyzed for MCS 1 and MCS 2.

Element	MCS 1 (wt.%)	MCS 2 (wt.%)
C	78.3	88.5
O	7.9	4.4
Fe	7.9	2.2
Si	4.8	3.9
Cu	1.1	0.9
Na	ND	0.1

ND—not detected. Values represent local semi-quantitative measurements obtained from selected microscopic regions and should not be interpreted as the bulk elemental composition of the magnetic colloidal suspensions.

**Table 2:** Previously reported physicochemical characteristics of the MIONPs used as precursor materials for the preparation of MCS 1 [18] and MCS 2 [19].

Precursor	Colloidal Suspension	Fuel	Post-Synthesis Treatment	Crystalline Phase	D_XRD_ [nm]	S_BET_ [m^2^/g]	D_BET_ [nm]	M_s_ [emu/g]	M_r_ [emu/g]	H_c_ [kA/m]
MIONPs_1	MCS 1	Citric acid monohydrate	None	Fe_3_O_4_	18	56	21	57.7	4.5	5.2
MIONPs_2	MCS 2	D-(+)- Glucose	H_2_O_2_ treatment	γ-Fe_2_O_3_	5	149	8	41.5	0.7	1.0

D_XRD_, average crystallite size calculated from X-ray diffraction data; S_BET_, specific surface area determined using the Brunauer–Emmett–Teller method; D_BET_, mean particle diameter estimated from BET data; M_s_, saturation magnetization; M_r_, remanent magnetization; H_c_, coercive field. Data were compiled from previously published studies and were not newly generated in the present investigation.

## Data Availability

Data is contained within the article. Further inquiries can be directed to the corresponding authors.

## Abbreviations

The following abbreviations are used in this manuscript:

AO	Acridine orange
ATCC	American Type Culture Collection
BET	Brunauer–Emmett–Teller
BF-STEM	Bright-field scanning transmission electron microscopy
DLS	Dynamic light scattering
DMSO	Dimethyl sulfoxide
EAU	European Association of Urology
EDX	Energy-dispersive X-ray spectroscopy
EMEM	Eagle’s Minimum Essential Medium
FBS	Fetal bovine serum
JC-1	5,5′,6,6′-Tetrachloro-1,1′,3,3′-tetraethylbenzimidazolylcarbocyanine iodide
MCS	Magnetic colloidal suspension
MIONPs	Magnetic iron oxide nanoparticles
MTT	3-(4,5-Dimethylthiazol-2-yl)-2,5-diphenyltetrazolium bromide
ND	Not detected
NRU	Neutral red uptake
PBS	Phosphate-buffered saline
PDI	Polydispersity index
PI	Propidium iodide
RCC	Renal cell carcinoma
SD	Standard deviation
SLS	Sodium lauryl sulfate
VSM	Vibrating-sample magnetometer
